# Manganese oxide nanomaterials: bridging synthesis and therapeutic innovations for cancer treatment

**DOI:** 10.1186/s40580-024-00456-z

**Published:** 2024-11-27

**Authors:** Sandip Gangadhar Balwe, Dohyeon Moon, Minki Hong, Joon Myong Song

**Affiliations:** College of Pharmacy, Seoul National University, Seoul, 08826 South Korea

**Keywords:** MONs, Anticancer material, TME, Hypoxia, Nanovaccine, Immunotherapy

## Abstract

The advent of precision medicine in oncology emphasizes the urgent need for innovative therapeutic strategies that effectively integrate diagnosis and treatment while minimizing invasiveness. Manganese oxide nanomaterials (MONs) have emerged as a promising class of nanocarriers in biomedicine, particularly for targeted drug delivery and the therapeutic management of tumors. These nanomaterials are characterized by exceptional responsiveness to the tumor microenvironment (TME), high catalytic efficiency, favorable biodegradability, and advanced capabilities in magnetic resonance imaging. These attributes significantly enhance drug delivery, facilitate real-time bioimaging, and enable early tumor detection, thereby improving the precision and effectiveness of cancer therapies. This review highlights the significant advancements in the synthesis and therapeutic applications of MONs, beginning with a comprehensive overview of key synthetic methods, including thermal decomposition, potassium permanganate reduction, exfoliation, adsorption–oxidation, and hydro/solvothermal techniques. We delve into the preparation of MONs and H–MnO₂-based nanomaterials, emphasizing their chemical properties, surface modifications, and toxicity profiles, which are critical for their clinical application. Moreover, we discuss the notable applications of H–MnO₂-based nanomaterials in pH-responsive drug release, overcoming multidrug resistance (MDR), immunotherapy, and the development of nanovaccines for synergistic cancer treatments. By addressing the current challenges in the clinical translation of MONs, we propose future research directions for overcoming these obstacles. By underscoring the potential of MONs to transform cancer treatment paradigms, this review aims to inspire further investigations into their multifunctional applications in oncology, thus ultimately contributing to more effective and personalized therapeutic strategies.

**Graphical abstract:**

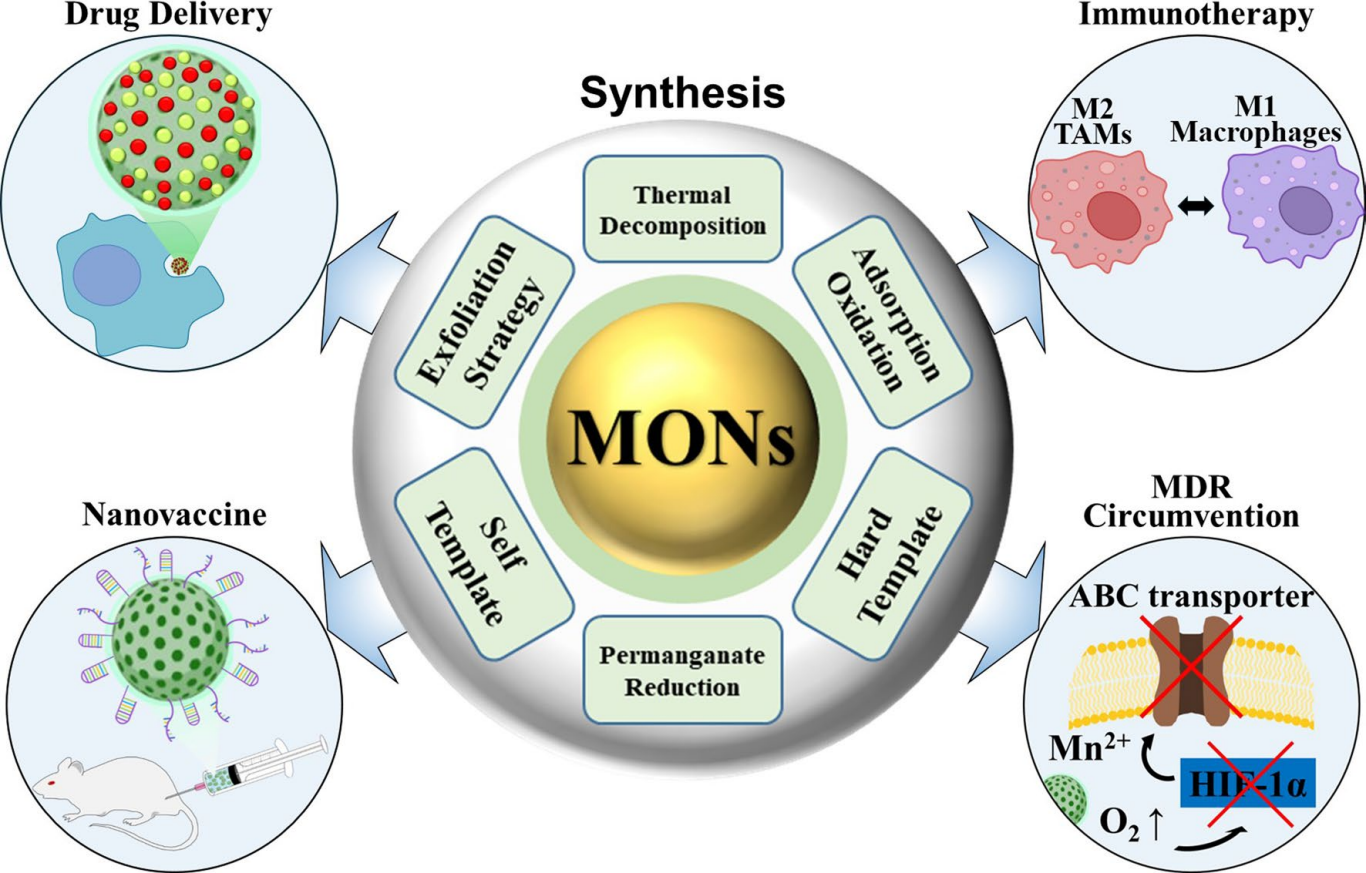

## Introduction

Cancer is a global health crisis and the leading cause of death worldwide, causing millions of deaths annually. Despite significant advancements in medical science, it remains one of the most pervasive and life-threatening diseases, with steadily increasing mortality rates [1, 2]. Cancer complexity is largely influenced by the tumor microenvironment (TME), which is characterized by abnormal tumor blood vessel architecture, elevated levels of hydrogen peroxide (H₂O₂), high concentrations of glutathione (GSH), and hypoxia [3–5]. These interrelated factors collectively impede the treatment efficacy and profoundly affect the success of conventional cancer therapies. This highlights the urgent need for innovative and effective treatment strategies that directly target the challenges posed by TME, to enhance both patient survival and quality of life [6, 7]. MONs have recently attracted significant attention in biomedicine because of their tunable morphology, excellent biocompatibility, and excellent catalytic properties [8, 9]. These nanostructures have made remarkable strides in various applications including biosensing, bioimaging, drug and gene delivery, and tumor therapy [10–12]. In particular, TME-responsive MnO₂-based nanoagents have emerged as a research hotspot owing to their simple fabrication processes, enhanced drug loading capacity, high specific surface area, controllable size and morphology, and ability to generate oxygen [13, 14]. MONs exhibit catalytic activities that convert hydrogen peroxide (H₂O₂) into oxygen (O₂) and manganese ions (Mn²⁺), as well as oxidizing glutathione (GSH) into oxidized glutathione (GSSG) [15]. These properties are particularly beneficial for addressing the TME, which is characterized by hypoxia, weak acidity, and elevated levels of GSH and H₂O₂ [16, 17]. By modulating immunosuppressive conditions, MONs enhance the effectiveness of immunotherapies and offer promising strategies for improving cancer treatment outcomes. MONs also significantly improved the efficiency of photodynamic therapy (PDT) by alleviating tumor hypoxia, further supporting anti-tumor immunotherapy [18, 19]. However, there is still a need to improve MONs for cancer cell targeting and synergistic therapy. Notably, hollow manganese dioxide nanoparticles (H-MnO₂ NPs) as synergistic nanovaccines remain an underexplored area with substantial potential for development [12]. Nanomaterial-mediated construction of nanovaccines presents exciting opportunities for cancer immunotherapy [20, 21]. By harnessing the unique properties of nanomaterials, these nanovaccines have the ability to significantly enhance the immune response against tumors [22, 23]. Moreover, the integration of immunotherapy with other treatment modalities, such as chemotherapy, radiotherapy, and targeted therapy, has become a prevalent strategy in cancer treatment [24, 25]. This approach improves therapeutic outcomes and addresses challenges such as immune resistance, making it a promising direction for the future of tumor immunotherapy. Nanoparticle (NP)-based vaccine delivery systems effectively overcome several limitations of traditional vaccines, including the instability of soluble macromolecular components, poor cellular internalization, and inefficient cross-presentation to cytotoxic T cells [26, 27]. Nanodelivery systems markedly enhance the efficiency of immunotherapy against tumor cells. Additionally, nanomaterials with inherent immunostimulatory properties can function as nanoadjuvants in tumor vaccines, promoting stronger immune responses and improving the overall therapeutic efficacy [28, 29]. This dual role of carriers and stimulants makes nanomaterial-based vaccines powerful tools for advancing cancer immunotherapy [30, 31]. Additionally, numerous studies indicate that H-MnO₂ NPs can regulate the immunosuppressive tumor microenvironment, transforming it into an immune-favorable condition [32]. O₂, the byproduct from the reaction between MnO₂ and H₂O₂ with H⁺, helps alleviate tumor hypoxia. As hypoxia inhibits anti-tumor immune responses by promoting the polarization of macrophages into M2 tumor-associated macrophages (TAMs), the ability to generate O₂ is a crucial strategy to overcome tumor immune evasion. It has been shown that MnO₂ NPs not only enhance innate immunity but also significantly improve adaptive immunity. Pro-inflammatory cytokines secreted by repolarized macrophages, innate immune responses, and high oxidative stress induce the release of tumor-associated antigens, which activate other immune cells, leading to the killing of tumor cells. This review provides an in-depth exploration of the synthesis and therapeutic applications of MONs in cancer therapy. While previous reviews have primarily focused on general applications of MONs [16, 33–35], this review specifically addresses the real-world challenges associated with translating MON-based therapies from preclinical studies to clinical practice. It first outlines the synthetic methods for MONs, followed by recent advancements in their use for TME targeting, immunotherapy enhancement, and the development of MON-based nanovaccines. Additionally, the review discusses practical barriers to the clinical implementation of MON-based therapies, offering insights into the challenges of scaling these therapies for real-world clinical use.

We begin by providing a comprehensive overview of essential synthetic methodologies, then focus on the preparation of H–MnO₂-based nanomaterials. Key aspects such as chemical properties, surface modifications, and toxicity profiles are highlighted. The review also examines the promising applications of H–MnO₂-based nanomaterials, including pH-responsive drug delivery systems, strategies to overcome multidrug resistance (MDR), advances in immunotherapy, and the development of nanovaccines aimed at enabling synergistic cancer treatments.

## Synthesis of MONs

In the last 20 years, various techniques have been developed to synthesize MONs in diverse forms and sizes, including stable oxides such as MnO, MnO₂, Mn₂O₃, Mn₃O₄, and MnOx [9, 36, 37]. These synthesis methods produce a wide array of morphologies, such as hollow nanoparticles, nanosheets, nanoflowers, nanorods, nanotubes, and honeycomb structures. Commonly employed synthetic strategies include thermal decomposition, potassium permanganate (KMnO_4_) reduction, exfoliation, adsorption–oxidation, and hydro/solvothermal techniques. Each method has unique strengths and limitations that affect the characteristics and potential applications of the produced MONs. Furthermore, a comparison of the main methods for synthesizing MONs is provided in Table 1.

**Table 1:** Overview of various synthesis methods for MONs

Synthetic Methods	Mn sources	Products	Advantages	Disadvantages	Ref.
Thermal decomposition method	MxCupx (M: metal ion; Cup: N-nitrosophenylhydroxylamine, C_6_H_5_N(NO)O^−^), Mn(II) acetate, [Mn(acac)_2_] (acac = acetylacetonate), Mn(II) formate, Mn(II) oleate.	MnO, Mn_3_O_4,_ Mn_2_O_3_	Good size control, well-defined morphology, narrow size distributions, high crystallinity of individual, dispersible nanocrystals, and the ability to produce various MnO morphologies.	Requires stringent oxygen-free, high-temperature conditions, producing pure-phase MnO nanocrystals is challenging, often resulting in oily solvent-capped products.	38–42
Potassium permanganates (KMnO_4_) reduction method	KMnO_4_	MnO_x,_ MnO_2_	Versatility in creating diverse MnO₂-based nanocomposite materials, ultra-fast aqueous-phase synthesis at ambient temperature, excellent dispersion in water, and the ability to produce various morphologies depending on the templates used.	Uncontrollable due to the rapid growth process.	43–48
Exfoliation Strategy	Mn(NO_3_)_2_ MnCl_2_, MnO_2_·nH_2_O	MnO_2_	Benefit of obtaining single-layer MnO₂ nanosheets (NSs).	Long reaction times, high temperatures, complex synthetic processes, uncontrollable particle sizes, and poor dispersion in water.	52–58
Adsorption–oxidation method	MnCl_2_	MnO_2,_ MnOx-SPIONs, C@SMn	Very narrow size distribution, aqueous-phase synthesis, and reactions at ambient temperature.	Requires adsorption of Mn^2+^ ions and alkali condition are necessary	59–61
Hydro/solvothermal method	MnCl₂·4 H₂O, Mn(II) acetate, MnSO₄·H₂O/KMNO_4_ Mn (II) phthalocyanine (Mn-Pc) Mn(II) stearate	MnO₂, Mn_3_O_4,_ Mn-carbon dots (Mn-CDs)	Narrow size distribution	Long reaction times and the need for specialized reaction vessels.	62–67

### Thermal decomposition method

**Fig. 1:**
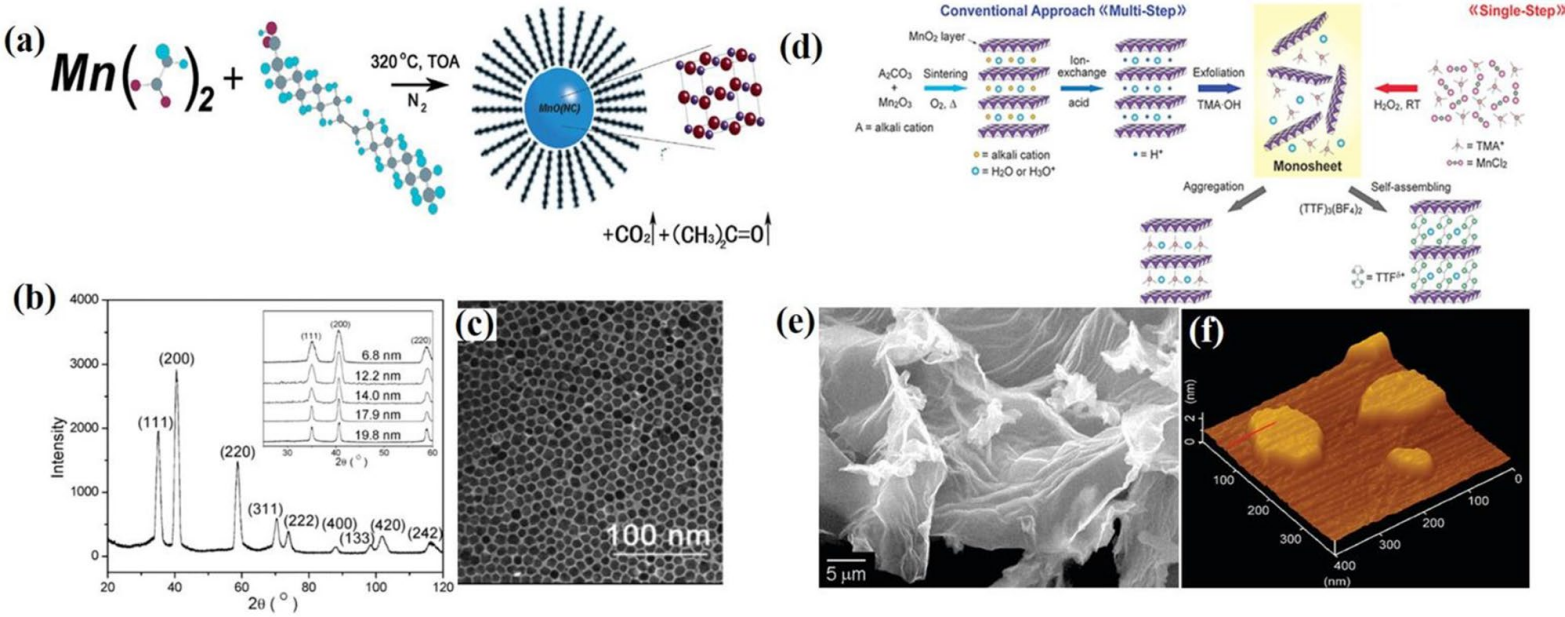
Synthesis methods for MONs. (**a**) Schematic diagram illustrating the synthesis procedure for MnOx nanocrystals via thermal decomposition. (**b**) X-ray powder diffraction (XRD) pattern of 7 nm diameter MnO nanocrystals. (**c**) TEM image of MnO nanocrystals. Reproduced with permission [39]. (**d**) Schematic illustration of the synthetic procedure for MnO₂ NSs via a multi-step or single-step exfoliation method. (**e**) SEM image of a freeze-dried aggregate of MnO₂ NSs. (**f**) Tapping-mode AFM image of MnO_2_ NSs. Reproduced with permission [56]

Thermal decomposition is one of the most popular methods for preparing monodisperse, highly crystalline, well-defined, and phase-pure high-quality nanocrystals with sizes less than 10 nm. Thermal decomposition is an oxygen-free organic-phase synthesis process that involves dissolving organic precursors in high-boiling organic solvents with the assistance of surfactants and decomposing the precursors at elevated temperatures. By using different manganese precursors and controlling various reaction parameters such as reaction time, solvent, and temperature, manganese dioxide NPs with different morphologies and sizes were prepared. Alivisatos et al. first reported a non-hydrolytic single-precursor approach for the synthesis of dispersable nanocrystals of manganese dioxide NPs [38]. The manganese Cupferron (N-nitrosophenylhydroxylamine, C_6_H_5_N(NO)O^−^) complex with the manganese ion coordinated *via* the oxygen atoms of the Cup ligand in a bidentate manner has proven to be a promising molecular precursor. A solution of manganese cupferron complex in octylamine was injected into trioctylamine at 360 °C for 10 min yielding monodisperse colloidal Mn_3_O_4_ NPs. The high temperature and complexity involved in the preparation of complex manganese cupferrons limit the use of this method. In 2003, O’Brien et al. synthesized monodispersed MnO nanocrystals with a length of 7 nm via the thermal decomposition of manganese acetate (Mn(CO_2_CH_3_)_2_) [39]. The synthesis scheme is illustrated in Fig. 1a. In this process, dry Mn(CO_2_CH_3_)_2_ was added to a reaction mixture containing trioctylamine and oleic acid at room temperature under a nitrogen atmosphere. The mixture was then rapidly heated to 320 °C for 1 h. After cooling, the mixture was extracted with hexane and precipitated with ethanol to yield uniform MnO nanocrystals with an efficiency of up to 80%. It is believed that the reaction proceeds through the decomposition of acetate to form a manganese oxide-oleic acid complex with the release of CO_2_ and acetone. Oleic acid served as a surfactant, whereas trioctylamine acted as an organic solvent to achieve high temperatures. The nanocrystals were characterized by X-ray powder diffraction (XRD) and transmission electron microscopy (TEM) (Fig. 1b and c). In conclusion, high-quality MnO nanocrystals with alkyl chain capping groups were successfully prepared and exhibited stability in nonpolar solvents. This synthetic procedure offers several advantages for the fabrication of transition metal oxide nanocrystals, including simplicity, reproducibility, and excellent yield. This method directly produces highly crystalline and monodisperse nanocrystals, eliminating the need for further size selection. In addition, the particle size can be easily and incrementally increased, facilitating kinetic studies.

In 2004, Park et al. developed a new method for the synthesis of monodisperse spherical Mn_3_O_4_ NPs using [Mn(acac)_2_] (acac = acetylacetonate) as a precursor [40]. They heated a slurry of [Mn(acac)_2_] in oleylamine (with a 1:24 molar ratio) at 180 °C under an argon atmosphere for 9 h. Transmission electron microscopy (TEM) images of the Mn_3_O_4_ NPs revealed highly crystalline and monodisperse spherical nanoparticles with diameters of approximately 10 nm. In this method, temperature control allowed the achievement of varying particle sizes of Mn_3_O_4_ NPs. Smaller Mn_3_O_4_ NPs, with a diameter of 6 nm, were obtained at 150 °C, whereas larger particles, with a diameter of 15 nm, were able to be prepared at 250 °C. The main advantage of this method is that it synthesizes only pure cubic MnO nanoparticles (NPs) by introducing 10 equivalents of water into the reaction slurry containing [Mn(acac)₂] in oleylamine (at a 1:24 molar ratio). The presence of water prevents further oxidation of the formed MnO, likely because water is involved in the decomposition of the acetylacetonate ligand, which acts as an oxygen source. Hyeon et al. (2004) reported a new protocol for the synthesis of ultralarge-scale monodisperse MnO nanocrystals using inexpensive and non-toxic metal salts as reactants [41]. They successfully synthesized up to 40 g of monodisperse nanocrystals using a single-step process. The reaction involved the formation of a metal-oleate complex using a metal chloride and sodium oleate, followed by the decomposition of this complex in a high-boiling solvent to yield monodisperse magnetic nanocrystals. The main advantage of this synthetic method is that it provides a highly efficient approach for the successful synthesis of monodisperse nanocrystals of various transition metal oxides through the thermolysis of metal-oleate complexes. In 2006, Whitmire et al. synthesized MnO nanocrystals using manganese formate (Mn(HCOO)₂) as a precursor in the presence of tri-n-octylamine and oleic acid [42]. The main advantage of this thermal decomposition method is its ability to produce various MnO morphologies, including flower-shaped, star-shaped, nanorod, barbell rod, hexapod, octapod, rhombohedral, and pseudo-hexagonal shapes, through precise control. Despite these advances, thermal decomposition requires rigorous oxygen-free high-temperature conditions. Additionally, it is challenging to produce pure-phase MnO₂ nanocrystals using this method. For biomedical applications of manganese oxides (MONs), it is necessary to translate oily solvent-capped nanocrystals into an aqueous phase.

### Potassium permanganates (KMnO_4_) reduction method

KMnO_4_, as a strong oxidant, can react with reductants to generate MONs. The KMnO_4_ reduction method is often performed in aqueous solutions at room temperature and the product is MnO_2_. In 2008, J. He et al. successfully prepared various monodisperse manganese oxide nanomaterials by treating a precursor initially prepared by mixing a KMnO₄ solution with oleic acid [43]. In this process, KMnO₄ was dissolved in water, and the mixture was stirred for 30 min at room temperature. Subsequently, oleic acid was slowly added to form a stable emulsion. After maintaining the emulsion at room temperature for some time, brown-black products were collected, washed with water and ethanol several times to remove impurities, and then dried in air at 80 °C. The precursor was finally calcined in air at 150 and 200 °C for 5 h. The phase structure of the manganese oxide nanostructure was controlled by treating the precursor at different temperatures, resulting in a transformation from a layered manganese oxide to a tetragonal hausmannite structure. The manganese oxide nanostructures were characterized using TEM, X-ray diffraction (XRD), scanning electron microscopy (SEM), and energy-dispersive spectroscopy (EDS). Wu et al. Their study reported a one-step synthetic protocol for reducing manganese from KMnO₄ to MnO₂ NPs using the cationic polyelectrolyte poly(allylamine hydrochloride) (PAH). This method is advantageous due to its rapidity, high reproducibility, and the provision of a stable MnO₂ colloidal suspension with an average nanoparticle size of 15 nm [44]. In this approach, PAH functions both as a reducing agent to convert KMnO₄ to MnO₂ and as a protective layer to stabilize the formed MnO₂ NPs through electrostatic repulsion. In the method, MnO₂ NPs were prepared by directly mixing aqueous solutions of KMnO₄ and PAH (15 kDa). The mixture was left at room temperature for 15 min until all of the permanganate was reduced to MnO₂ NPs. The formation of MnO₂ NPs was confirmed by UV-visible absorption spectroscopy. Subsequently, the MnO₂ NPs were washed three times with deionized water using ultracentrifugation (50,000 rpm for 1 h) to obtain approximately 15 nm MnO₂ NPs stabilized with PAH. Liu et al. reported a new method for reducing KMnO₄ using 2-(*N*-morpholino)ethanesulfonic acid (MES) buffer to obtain amorphous MnO₂ nanosheets on upconversion nanoparticles (UCNPs) [45]. In this method, the core-shell NaYF₄/Tm@NaYF₄ nanoparticles were first synthesized and added to a microcentrifuge tube containing MES buffer. Various amounts of KMnO₄ were then introduced into the tube. The mixture was sonicated for 30 min until a brown colloid formed. The MnO-modified UCNPs were subsequently collected by centrifugation, washed three times with deionized water to remove excess potassium and free manganese ions, and redispersed in deionized water. The formation of upconversion nanoparticle/MnO₂ nanosheet assemblies, stabilized by electrostatic interactions, was confirmed using TEM. In 2017, Liu et al. demonstrated that MnO₂ could be effectively coated onto the surface of freshly prepared silica NPs by mixing them with KMnO₄. The KMnO₄ was reduced by the unreacted organosilica present on the silica NPs, leading to the formation of a MnO₂ layer [46].

Similarly, diverse MnO₂-based NPs can be prepared by reacting KMnO₄ with various reductants, such as graphene oxide, carbon dots (CDs), Na₂S₂O₃, and hydroxylated polyethylene glycol (PEG) [47–51]. In conclusion, the KMnO₄ reduction method is an excellent approach for preparing a variety of MnO₂ NPs. It offers the advantages of ultrafast aqueous phase synthesis at room temperature, versatility in creating diverse MnO₂-based nanocomposite materials, and the ability to achieve different morphologies based on the templates used.

### Exfoliation strategy

The exfoliation strategy involves the disintegration of layered compounds or bulk materials into their constituent single layers, thus making it an effective and important method for obtaining 2D materials. In the biomedical field, 2D materials have garnered significant attention and are widely used in applications such as drug and gene delivery, biosensing, and tumor therapy, owing to their fascinating physicochemical properties. In the early 2000s, Ooi et al. first observed the swelling and delamination behavior of birnessite-type manganese oxide upon intercalation with tetraalkylammonium ions [52]. In this process, BirMO(H) was synthesized by the acid exchange of highly crystalline sodium birnessite. The intercalation of tetraalkylammonium ions was studied. BirMO(H) was soaked in tetraalkylammonium hydroxide solutions of varying concentrations for 7 d at 298 K. The amount of tetraalkylammonium hydroxide used ranged from 0.5 to 25 times the exchangeable capacity of BirMO(H). After soaking, the solid was filtered through a membrane filter, and the filtrates were washed with water and analyzed using XRD. The intercalation of tetraalkylammonium ions into BirMO(H) occurred *via* an ion-exchange mechanism. Delamination followed water washing of the TMA^+^-intercalated samples because of the decreased surface charge density and reduced ion concentration in the interlayer. Air-drying of the delaminated solid leads to irreversible reassembly of the manganese sheets, resulting in a layered compound with dehydrated TMA^+^ ions in the interlayer. The swelling and delamination properties are influenced by the electrostatic attractive forces between the interlayer cations and anionic sheets, repulsive forces from the hydration of the interlayer cations, and attractive forces due to hydrogen bonding. In 2001, Suib et al. utilized two types of organic amine/ammonium species (tetraalkylammonium hydroxides (TAA) and diamines (DA)) to intercalate birnessite H-MnO_2_ and synthesize a nanometer-sized H-MnO_2_ layer [53]. In this process, potassium manganese oxide materials with a synthetic birnessite structure, designated as K-OL-1, were synthesized using reduction methods. K-OL-1 was then ion-exchanged with H^+^ to produce H-OL-1, which was utilized in subsequent intercalation reactions. Various tetraalkylammonium hydroxides (TAA), including tetramethylammonium hydroxide, tetraethylammonium hydroxide, tetrapropylammonium hydroxide, and tetrabutylammonium hydroxide, as well as diamines (DA), such as ethylenediamine, 1,6-diaminohexane, and 1,10-diaminooctane, were used for the intercalation of H-OL-1. The samples characterized using XRD, elemental analysis, UV-visible spectroscopy, EDX, and TEM. In 2003, T. Sasaki et al. synthesized unilamellar two-dimensional crystallites of MnO₂ with a thickness of approximately 0.77 nm through the delamination of layered manganese oxide [54]. In this method, a layered manganese oxide, K₀.₄₅MnO₂, was first prepared by calcining a stoichiometric mixture of KOH and Mn₂O₃ at 1073 K for 60 h under an O₂ gas flow. The resulting K₀.₄₅MnO₂ was then subjected to acid digestion for 10 days to remove the interlayer K⁺ ions completely. The resulting solid, H₀.₁₃MnO₂·0.7 H₂O, was washed several times with water and then air-dried. Treatment with aqueous solutions of tetrabutylammonium hydroxide (TBAOH) at various concentrations led to intercalation, osmotic swelling, and delamination into single sheets. In 2007, Sasaki et al. reported the synthesis and delamination of manganese oxide nanobelts with birnessite-type layered structures [55]. K-birnessite nanobelts (K ≤ ₀ ≥.₃₃MnO₂·0.5 H₂O) were characterized by a length of several tens of micrometers, a width of hundreds of nanometers, and a thickness of approximately 15 nm. They were synthesized by hydrothermally treating a KMnO₄–MnCl₂ mixture in a highly concentrated KOH aqueous solution. The K-birnessite nanobelts were converted to H-birnessite (H ≤ ₀ ≥.₀₈MnO₂·0.7 H₂O) by treatment with an aqueous solution of (NH₄)₂S₂O₈ while retaining their high crystallinity and belt-like morphology. The swelling and delamination behaviors of H-birnessite nanobelts in the aqueous solutions of quaternary ammonium hydroxides were studied. In TBAOH solutions, H-birnessite exhibited limited swelling and delamination. However, the compound underwent significant osmotic swelling in the TMAOH solution. Water washing of the TMAOH-treated samples greatly enhanced the degree of swelling, while preserving the three-dimensional crystalline order. Upon further contact with TBAOH solution, the highly swollen phase was predominantly delaminated into unilamellar nanosheets. These nanosheets retained the long-axis morphology of the parent nanobelts and exhibited lateral sizes on the micrometer scale. The colloidal suspension of the nanosheets displayed an optical absorption band at approximately 380 nm, which is in marked contrast to the relatively constant and featureless UV-to-visible light absorption of birnessite prior to delamination. In 2008, Yoshida et al. demonstrated a simple, rapid, and facile single-step approach to directly produce MnO₂ monosheets through the chemical oxidation of Mn²⁺ ions in the presence of tetramethylammonium cations in an aqueous solution at room temperature [56]. The schematic illustration of the synthetic procedure for MnO₂ nanoshells (NSs) *via* either multi-step or single-step processes is depicted in Fig. 1d. In this method, lithium- and sodium-type birnessites were first prepared using hydrogen peroxide (H₂O₂) as an oxidizing agent for Mn²⁺ ions. Along with H₂O₂, TMAOH in an aqueous MnCl₂ solution was utilized, resulting in a dark brown suspension in the open air at room temperature. The obtained MnO₂ NSs were characterized by SEM and tapping-mode AFM, and the formation of single-layer MnO₂ NSs was confirmed with an approximate thickness of 0.9 nm (Fig. 1e and f). This one-step approach yields a colloidal suspension of MnO₂ monosheets with excellent efficiency and a processing time of less than a day.

**Fig. 2:**
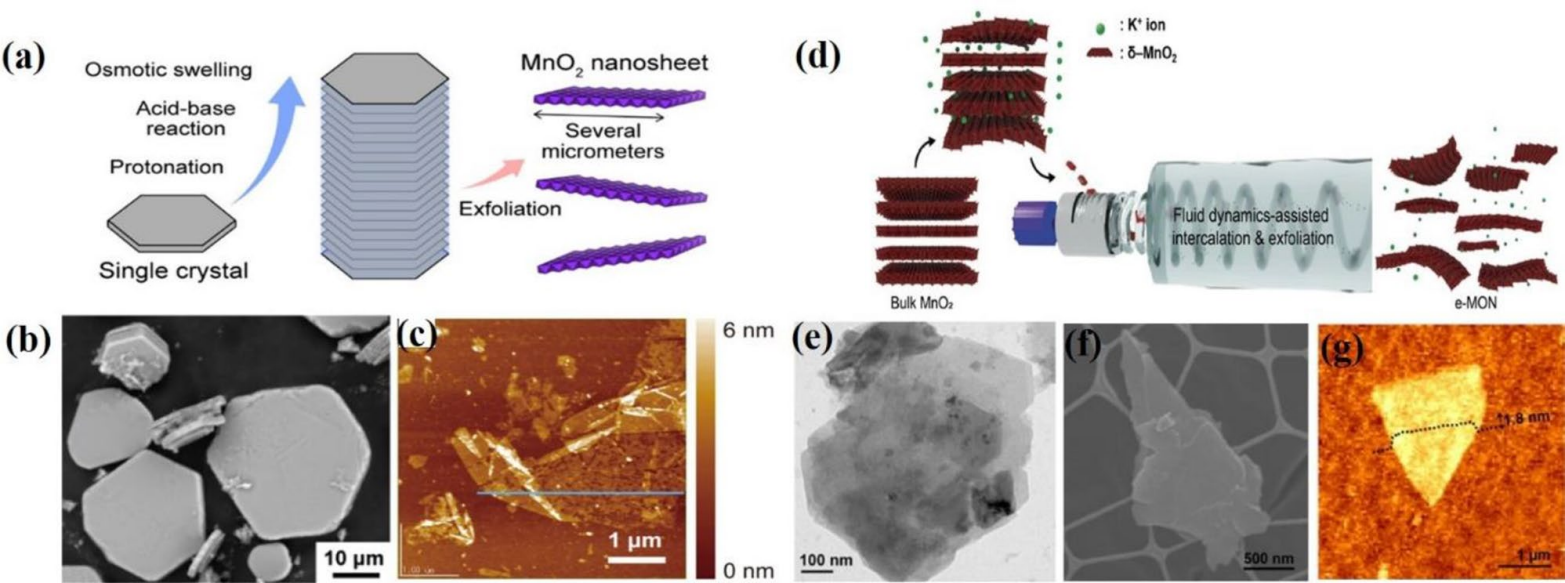
Exfoliation strategies for MnO₂ nanosheets (NSs). (**a**) Schematic diagram illustrating the synthesis procedure for MnO_2_ NSs. (**b**) SEM images of MnO_2_ NSs (**c**) AFM image of MnO_2_ NSs obtained from the protonated single crystals. Reproduced with permission [57]. (**d**) Schematic illustration of the synthesis of e-MON using a fluid-dynamic assisted intercalation and exfoliation method. (**e**) HR-TEM image of e-MON. (**f**) SEM image of e-MON. (**g**) AFM image of e-MON. Reproduced with permission [58]

Recently, Fujimoto et al. successfully developed large-sized hexagonal K_*x*_MnO_2_ single crystals with a lateral size of 50 μm and a thickness of 10 μm using the flux method [57]. The schematic illustration of the synthetic procedure for MnO₂ NSs by a single-step process is shown in Fig. 2a K_2_MoO_4_ was employed as the flux source and polycrystalline K_0.45_MnO_2_ served as the seed crystal. These K_*x*_MnO_2_ single crystals were used to prepare large MnO_2_ nanosheets using a process designed to minimize cracking during soft chemical delamination. The reaction of K_*x*_MnO_2_ with (NH_4_)_2_S_2_O_8_ yields crystals with minimal cracking. Notably, these protonated crystals exhibit significant swelling when exposed to a TMAOH solution, with their thickness increasing from approximately 10 μm to 100 μm a tenfold expansion due to hydration-induced swelling in the TMAOH solution. Subsequent treatment of the swollen crystals with TBAOH facilitated their exfoliation into unilamellar MnO_2_ nanosheets, each with a lateral size of several micrometers and a thickness of approximately 1.2 nm. The MnO₂ nanosheets were characterized by SEM. AFM images of MnO₂ NSs were obtained from protonated single crystals (Fig. 2b and c). In 2022, Choi et al. reported a fluid-dynamics-assisted method for alkali cation intercalation and exfoliation to prepare exfoliated MnO₂ nanosheets (e-MON) from bulk MnO₂ [58]. A schematic illustration of the preparation of e-MONs using a fluid dynamics-assisted intercalation and exfoliation method is shown in Fig. 2d. This method produced ultrathin 2D MnO₂ nanosheets with a large surface area and a nanoporous structure. The exfoliation process was achieved using Taylor–Couette flow, which provides high shear and mixing behavior that facilitates the intercalation of potassium ions into the interlayer spaces of MnO₂, leading to the delamination of MnO₂ into mono- and few-layer nanosheets. In this approach, a mixture of synthesized δ–MnO₂ (ranging from 0.1 to 10 mg mL⁻¹) and potassium hydroxide (0.01 M in deionized water) was injected into the fluid-dynamic reactor and exfoliated at 2000 rpm for 1 h. Following exfoliation, the nanosheets were collected and purified by centrifugation at 5000 × *g* for 150 min, followed by secondary centrifugation at 450 × *g* for 150 min to remove residual ions, very small e-MON, and unexfoliated δ–MnO₂. The resulting e-MON dispersion was filtered and washed several times with deionized water. The e-MON powder was obtained by freeze-drying for 24 h and characterized by SEM, TEM, and AFM (Fig. 2e and g). Although significant progress has been made in the exfoliation method, it still has drawbacks including tedious synthetic processes, long reaction times, high temperatures, uncontrollable particle sizes, and poor dispersion in water.

### Adsorption–oxidation method

The adsorption–oxidation method for preparing MONs involves two steps: (1) Mn^2+^ ions are adsorbed *via* electrostatic interactions and (2) the adsorbed Mn^2+^ ions are oxidized to MnO_2_ in situ by oxygen under alkaline conditions at room temperature. The representative equations are as follows:

$$2\text{M}\text{n}{\text{Cl}}_{2}+4{\text{NaOH}}+{\text{O}}_{2}\to\:2{\text{M}\text{nO}}_{2}+4{\text{NaCl}}+4{\text{H}}_{2}\text{O}$$

**Fig. 3:**
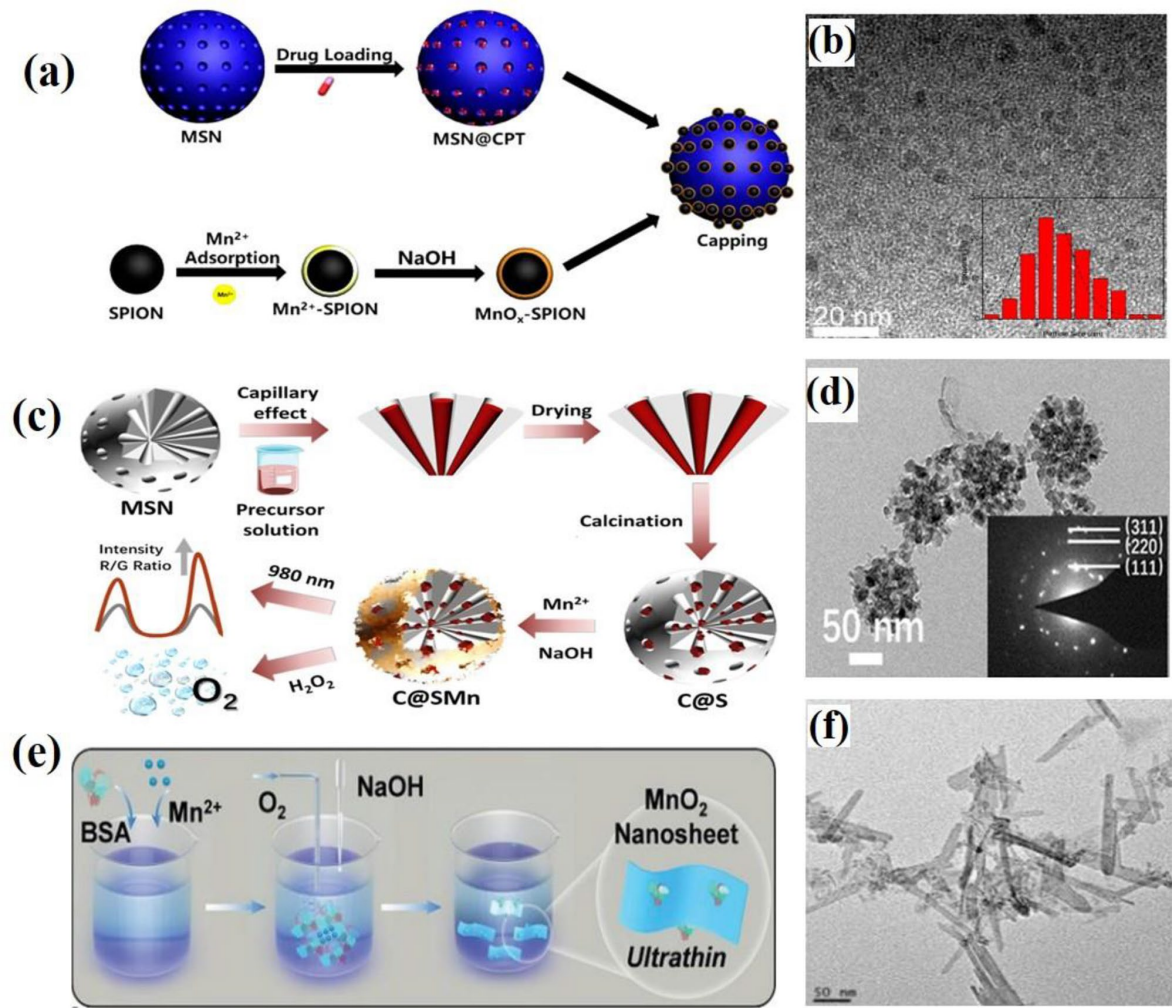
Schematic illustration of synthesis procedures using the adsorption–oxidation method. (**a**) Synthesis of MnOx-SPION capped MSN. (**b**) TEM images of MnOx-SPION. Reproduced with permission [59]. (**c**) Synthesis process of C@SMn. (**d**) TEM images of C@SMn. Reproduced with permission [60]. (**e**) Wet-chemical synthesis of enzymatic 2D MnO₂ nanosheets (NSs). (**f**) TEM images of MnO₂ NSs. Reproduced with permission [61]

Miao et al. employed an adsorption-oxidation method to synthesize ultrathin MnO_2_ layers coated on superparamagnetic iron oxide nanoparticles (SPIONs) [59]. A schematic illustration of the synthesis of MnOx-SPION MSN is shown in Fig. 3a. The process begins with the synthesis of monodisperse γ-Fe_2_O_3_ SPIONs with a uniform particle size of 4 nm. Iron(III) acetylacetonate was dissolved in triethylene glycol (TEG) and stirred for 1 h at 140 °C until a transparent solution is obtained. The temperature is then rapidly increased to 200 °C and maintained for 30 min, resulting in a brownish slurry of SPIONs. SPIONs were separated using acetone and washed with water and ethanol. Next, the SPIONs were dispersed in a saturated citric acid solution to modify their surfaces with-COOH groups. The resulting SPION-COOH was neutralized with a 2 M NaOH solution. A saturated MnCl_2_ solution was then added dropwise to the SPION-COOH dispersion until a brown transparent precipitate was formed, indicating the complete adsorption of Mn^2+^ onto the SPION surface. The Mn^2+^ adsorbed SPIONs were separated by centrifugation, redispersed in water, and treated with 2 M NaOH solution. After stirring for 2 h to complete the oxidation of the manganese hydroxide, an ultrathin MnOx layer was coated onto the SPION surface. Finally, a citric acid solution was added to further modify the MnOx-SPIONs with carboxylic acid groups. The presence of abundant carboxylate groups on the SPION surface facilitated the adsorption of manganese ions, enabling the formation of an ultrathin manganese oxide layer (MnOx-SPIONs). The morphology and size of MnOx-SPIONs were confirmed by TEM analysis (Fig. 3b). Liu et al. utilized an adsorption-oxidation method to produce thin MnO_2_ layers coated on CaF_2_ and Er@silica (C@S) nanoparticles [60]. A schematic representation of the synthesis of C@SMn is shown in Fig. 3c. In this method, Mn^2+^ ions were incorporated into the lattices of CaF_2_ and Er silica crystals. To embed CaF_2_ and Er nanocrystals within the pores of mesoporous silica nanoparticles (MSN), a precursor solution was prepared by dissolving Ca(OAc)_2_, Yb(OAc)_3_, and Er(OAc)_3_ in deionized water and adding TFA, followed by stirring for 24 h. The MSN and the precursor solution were then mixed and allowed to immerse under slow stirring at 35 °C for 24 h. The resulting nanoparticles were centrifuged at 8000 rpm for 5 min and left at room temperature for 4 h. After air-drying at 80 °C overnight, the nanoparticles were further calcined at 600 °C for 3 h to obtain C@S NPs. Subsequently, MnCl_2_ and C@S were dissolved in deionized water at various weight ratios, and the mixture was stirred for 30 min at room temperature, followed by the addition of a 0.05 M NaOH solution. The mixture was then stirred for 2 h. Finally, the CaF_2_,Er@silica@MnO2 (C@SMn) core-shell nanoparticles were collected by centrifugation and washed three times with ethanol. The morphology of C@SMn NPs was characterized by TEM (Fig. 3d). Recently, Xie et al. reported a novel one-step wet-chemical method for the controlled synthesis of MnO_2_ NSs using a protein-directed adsorption–oxidation approach as shown in Fig. 2e [61]. Using this method, the size and thickness of the nanosheets could be easily adjusted by varying the ratio of bovine serum albumin (BSA) to Mn^2+^ ions. BSA directs the nucleation of MnCl_2_ and Mn^2+^ is spontaneously oxidized by oxygen in alkaline solutions to form nanosheets. MnCl_2_ and a specified amount of BSA were dissolved in ultrapure water and stirred for 1 h at room temperature. Next, a NaOH solution was added to adjust the pH to approximately 10. The mixture was stirred overnight. Subsequently, the MnO_2_ NSs were collected via centrifugation and washed three times. The resulting MnO_2_ NSs were characterized by TEM (Fig. 3f). The main advantage of the adsorption–oxidation method is that it allows aqueous-phase synthesis reactions at ambient temperature, resulting in nanoparticles with a narrow size distribution. However, the effectiveness of this method is limited by the requirements for Mn^2+^ ion adsorption and alkaline conditions.

### Hydro/solvothermal method

**Fig. 4:**
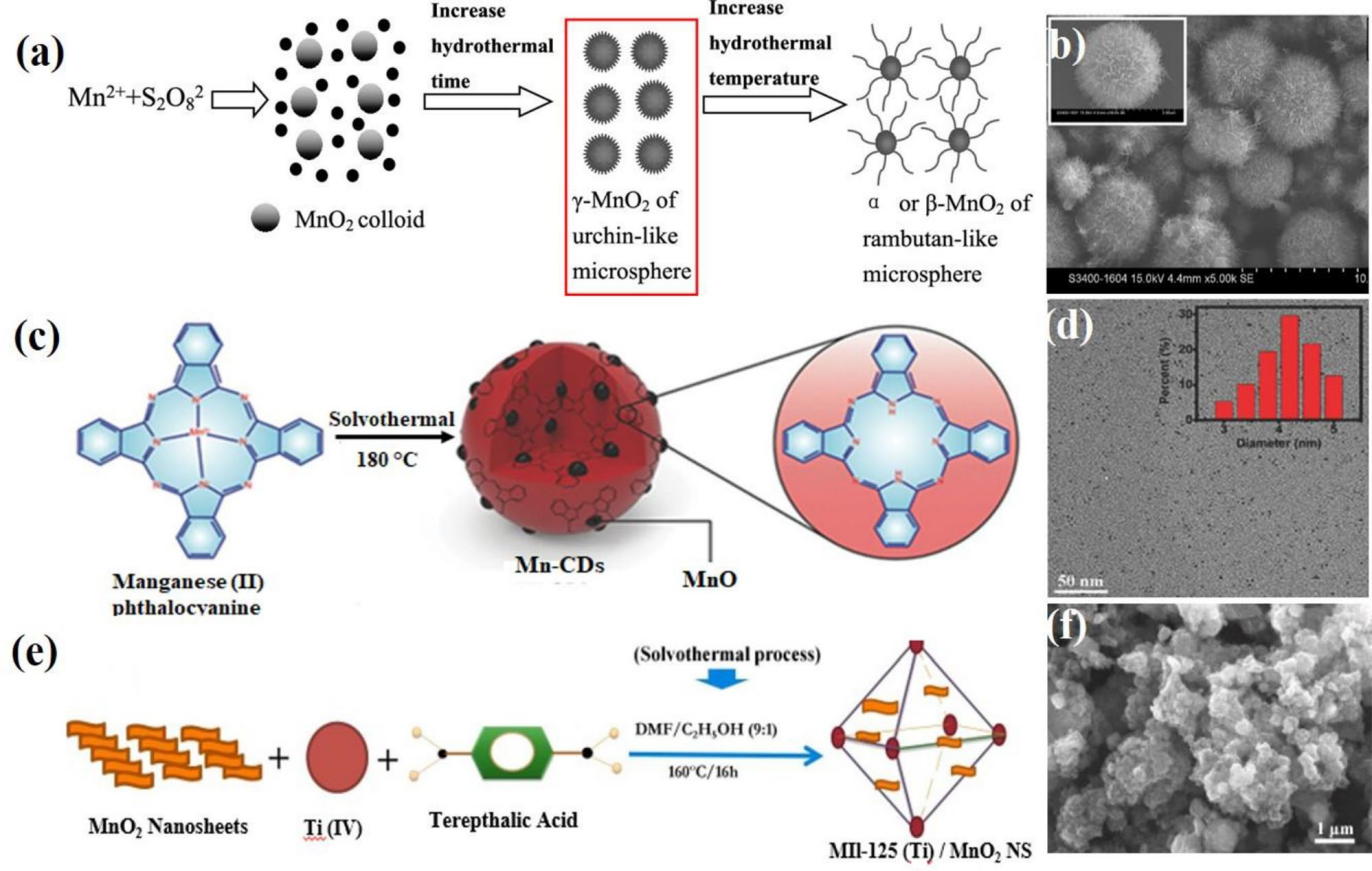
Schematic illustration of synthesis procedures using the hydro/solvothermal method. (**a**) Hydrothermal synthesis of α or β-MnO₂ microspheres. (**b**) SEM images of MnO_2_ microspheres. Reproduced with permission [63]. (**c**) Solvothermal method for the synthesis of Mn-CD. (**d**) TEM images of Mn-CDs. Reproduced with permission [65]. (**e**) Synthesis of MIL-125 (Ti)/MnO₂ NSs through a solvothermal process. (**f**) SEM images of MIL-125(Ti)/MnO_2_ NSs. Reproduced with permission [67]

The hydro/solvothermal method is a prominent synthesis process for the fabrication of nanomaterials. This technique employs a Teflon-lined autoclave, which is a specialized reaction vessel that contains various solvents to enhance solubility and reactivity under high-pressure conditions. Li et al. first reported the synthesis of α and β MnO_2_ single crystal nanowires using this hydrothermal method, which involves the oxidation of Mn^2+^ by S_2_O_8_^2−^, without the need for catalysts or templates [62]. Equal amounts of ammonium persulfate ((NH_4_)_2_S_2_O_8_) and manganese sulfate (MnSO_4_) were mixed with distilled water at room temperature to create a homogeneous solution. This solution was then transferred to a Teflon-lined stainless steel autoclave, sealed, and maintained at 120 °C for 12 h. After the reaction was completed, the resulting black solid was filtered, washed with distilled water, and air-dried at 120 °C. The micro- and nanostructures of the products were further examined by TEM. The advantages of this method include the low-temperature synthetic route based on simple reactions that do not require catalysts or templates. Additionally, it does not require expensive or precise equipment, ensuring a higher purity of the products while significantly reducing production costs. Thus, this method offers great potential for the scaling up of nanostructured materials. Similarly, the Yu group reported the synthesis of MnO₂ microspheres using a hydrothermal method with MnCl₂·4 H₂O, Mn(CH₃COO)₂·4 H₂O, and MnSO₄·H₂O as precursors [63]. The synthesis scheme is illustrated in Fig. 4a. In this method, MnCl₂·4 H₂O, Mn(CH₃COO)₂, and MnSO₄·H₂O were separately added to (NH₃)₂S₂O₈ and dissolved in deionized water. The resulting solutions were transferred into a Teflon-lined autoclave and heated at 80 °C for 4 h. The solid products were washed with deionized water until neutral pH, filtered, and dried at 80 °C. Notably, the morphology and crystallinity of MnO₂ were influenced by both the hydrothermal time and temperature. The nanoneedles gradually lengthened as reaction time and temperature increased, and the crystalline structure of MnO₂ shifted from γ-MnO₂ to α- and β-MnO₂ with rising hydrothermal temperatures. The obtained MnO₂ materials were characterized by SEM (Fig. 4b). Yue et al. developed a solvothermal route for the synthesis of single-crystalline α-MnO₂ nanowires with diameters ranging from 10 to 30 nm and lengths extending to several micrometers [64]. In this method, Mn₂O₃ powder was dispersed in an aqueous KOH solution and then transferred into a Teflon (PTFE)-lined autoclave. The autoclave was heated in an oven at 180 °C for 12 h to 1 week. After the hydrothermal treatment was performed, the precipitate was filtered and washed repeatedly with dilute HCl and deionized water. The final product was obtained by filtration and air drying. The morphology and crystal structure of the final product were characterized using SEM and HRTEM. This synthesis method for manganese dioxide nanowires is simple and efficient, requiring no catalyst to provide energetically favorable sites for the adsorption of reactant molecules and no template to direct nanowire growth.

Wang et al. employed a solvothermal approach to synthesize magnetofluorescent Mn-carbon dots (Mn-CDs) using Mn (II) phthalocyanine (Mn-Pc) as the precursor in absolute ethyl alcohol, as shown in Fig. 4c [65]. In this method, Mn-Pc was first dispersed in absolute ethyl alcohol via ultrasonication for 30 min. The dispersion was then heated at 180 °C for 24 h in a Teflon-lined autoclave. After cooling to room temperature, the resulting Mn-CDs were filtered to remove larger particles and dialyzed multiple times against ethyl alcohol to eliminate any residual free Mn-Pc. The obtained Mn-CDs were characterized using TEM (Fig. 4d). Ji et al. developed a two-phase route for the shape- and size-controlled synthesis of monodisperse Mn_3_O_4_ nanocrystals capped with organic ligands and dissolved in a nonpolar solvent to ensure excellent stability [66]. By carefully varying the reaction conditions, nanocrystals can be engineered into either relatively monodisperse spherical shapes or nearly perfect cubes. In this approach, toluene and water were used as solvents. Manganese(II) stearate (Mn(SA)₂) and *tert-butylamine* served as sources of manganese and hydroxide, respectively. Dodecylamine in toluene acts as a ligand to stabilize the nanocrystals. To synthesize these nanocrystals, a mixture of Mn(SA)_2_, dodecylamine, and toluene was loaded into a test tube and heated to 80–100 °C to form an optically clear solution. After cooling to room temperature, the solution was mixed with aqueous tert-butylamine and heated in an autoclave without stirring. The autoclave was then sealed and maintained at 180 °C for 4 h. The resulting crude Mn_3_O_4_ nanocrystals were precipitated with methanol and isolated via centrifugation. The reaction temperature significantly influenced the shape of the nanocrystals. At a relatively low temperature of 120 °C, the Mn_3_O_4_ nanoparticles exhibited cubic shapes with an average particle size of 7.9 nm, as determined using HRTEM. Recently, Majid’s group employed a solvothermal approach for the synthesis of MIL-125(Ti)/MnO₂ NSs [67]. The synthetic method for MIL-125 (Ti)/MnO₂ NSs through this solvothermal process is depicted in Fig. 4e. In this method, the MnO₂ NSs were sonicated in DMF. In a separate reaction vessel, DMF and ethanol were mixed with terephthalic acid, followed by the addition of the MnO₂ NS dispersion. Titanium isopropoxide was then added dropwise, and the mixture underwent a solvothermal step in a Teflon-lined autoclave at 160 °C for 16 h. The resulting yellow product was washed with DMF and ethanol and then dried in an oven at 100 °C. The obtained MIL-125(Ti)/MnO₂ NSs were characterized using SEM (Fig. 4f).

It is important to highlight that each MON synthesis method comes with its own set of unique strengths and limitations. The thermal decomposition method excels at precisely controlling the particle size and morphology, but its reliance on oily solvents can create surface capping issues that limit its biomedical applicability. The permanganate reduction method is widely employed to fabricate MnO-based composite materials, allowing diverse morphological variations. However, the exfoliation strategy effectively yields single-layer MnO₂ nanosheets, although it often requires complex procedures and can result in inconsistent particle sizes. Other synthetic approaches have also been reported, including one-pot microwave synthesis and polyol methods, which have further expanded the toolkit available for producing manganese oxide nanomaterials in the field of nanomedicine.

### Synthesis of hollow MnO_2_ nanomaterial (H-MnO_2_)

**Fig. 5:**
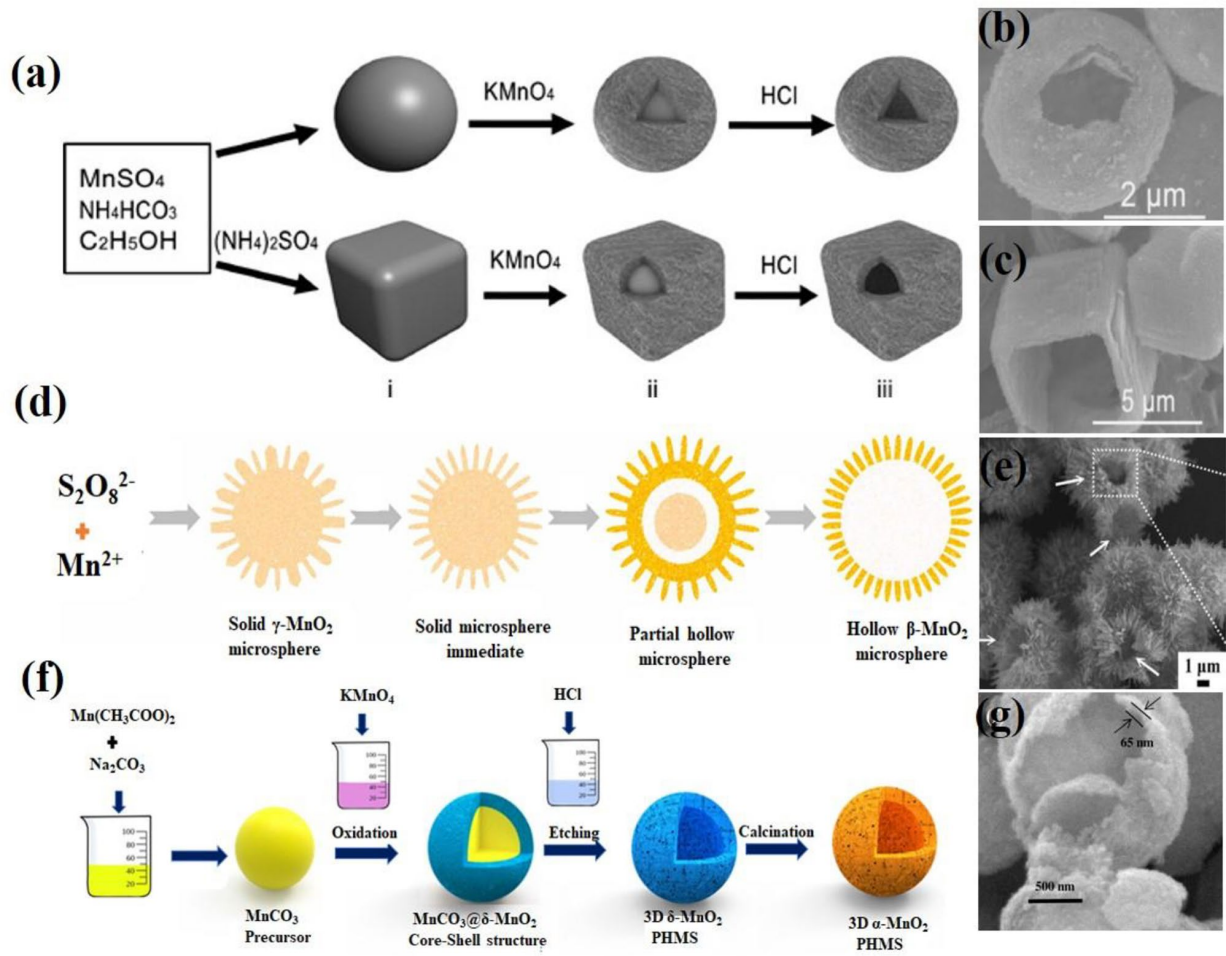
Schematic illustration of the facile synthesis of MnO₂ hollow nanostructures *via* a self-templated method. (**a**) Diagram of the fabrication of MnO₂ hollow nanostructures (i: MnCO_3_, ii: MnO_2_ shell structures with MnCO_3_ cores, iii: MnO_2_ hollow morphologies). (**b**,** c**) SEM images of MnO_2_ hollow microspheres and microcube. Reproduced with permission [70]. (**d**) Formation process of hollow β-MnO₂ microspheres. (**e**) SEM image of hollow β-MnO_2_ microspheres. Reproduced with permission [71]. (**f**) Synthesis of 3D α-MnO₂ PHMSs. (**g**) SEM image of α-MnO_2_ PHMSs. Reproduced with permission [74]

Hollow nanomaterials, distinguished by their cavity-like structures at the nano- or micro-scale, have gained considerable attention owing to their unique properties, including high surface area, low density, and excellent biocompatibility [68]. These materials typically feature thin and porous shells enclosing an internal void. This feature makes them highly promising for diverse applications such as catalysis, energy storage, sound insulation, controlled drug release, targeted drug delivery, and the simultaneous treatment of cancers when the surface or core of hollow nanomaterials is functionalized with specific ligands or nanoparticles [69]. In the biomedical field, hollow nanostructures with mesoporous shells and large cavities are exceptional for drug and gene delivery because they can carry significant quantities of therapeutic agents. The release of these agents can be precisely regulated by modifying the shell structure or by applying specialized coatings, making hollow nanomaterials highly effective in advanced drug delivery systems. Recently, H-MnO₂ NPs have been extensively studied as potential oxygen-supplying materials due to their unique characteristics, such as high sensitivity to H₂O₂ and H⁺, both of which are abundant in the TME. These properties make H-MnO₂ NPs promising candidates for applications in cancer therapy and related biomedical fields. In this section, recent progress in the design, synthetic strategies, and functionalization of H-MnO₂ NPs and MnO₂-based nanocomposite materials is discussed. According to their formation mechanisms, the synthesis routes for H-MnO₂ NPs can be broadly categorized into two main approaches: (I) self-templated synthesis and (II) hard templating synthesis. The self-templated synthesis involves the spontaneous transformation of MnO₂ precursors into hollow structures without the use of external templates. Self-templated approaches often rely on processes such as the Kirkendall effect, Ostwald ripening, or selective etching, in which the internal material dissolves or migrates, leaving behind a hollow structure. This strategy is advantageous because of its simplicity and cost-effectiveness, as no external templates are required. In 2008, Li et al. first reported the controlled synthesis of MnO₂ hollow nanostructures using a self-assembly method with an intermediate crystal-templating process [70]. The three-step synthesis process is illustrated in Fig. 5a. Initially, MnCO₃ microspheres were prepared *via* a simple mixing method. In the second step, MnO₂ hollow microspheres were formed by using solid MnCO₃ crystals as a template and mixing them with varying amounts of KMnO₄. In the final step, the MnCO₃ core was etched away with HCl. The resulting product was obtained by centrifugation and repeatedly washed with ultrapure water. The obtained MnO_2_ hollow microspheres and microcube were characterized by SEM (Fig. 5b and c). Yu et al. developed a novel self-templated method for synthesizing hollow β-MnO₂ microspheres, which involved the phase transformation of γ-MnO₂ to β-MnO₂ [71]. In this process, Na₂S₂O₈ and MnSO₄·H₂O were added to an H₂SO₄ solution and stirred vigorously for 30 min. The resulting colorless solution was sealed in a 50 mL Teflon-lined autoclave and heated to 140 °C for 12 h. Subsequently, the black solid product was thoroughly washed with deionized water and ethanol to remove impurities and then air-dried at 60 °C overnight. The study highlighted the critical role of reaction temperature and H₂SO₄ concentration in determining the phase and morphology of the final product. At 140 °C, the phase transformation to β-MnO₂ occurred, while further increasing the temperature to 160–180 °C led to the formation of uniform β-MnO₂ nanorods. This work not only presents an efficient method for preparing hierarchical hollow MnO₂ crystals but also provides valuable insights into their growth mechanism.

A self-templated mechanism involving phase transformation can explain the formation of hierarchical hollow β-MnO₂ microspheres. Solid γ-MnO₂ microspheres serve not only as templates for hollow microsphere formation but also as precursors for their transformation into β-MnO₂ (Fig. 5d). The growth process begins in step I, where S₂O₈²⁻ reacts with Mn²⁺ to form solid γ-MnO₂ microspheres within the synthesis system. In step II, further hydrothermal treatment induces the outer nanosheets to transform into nanorods while the γ-MnO₂ structure begins to convert into β-MnO₂. This transformation occurs because γ-MnO₂ is metastable in acidic conditions and gradually changes to the more thermodynamically stable β-MnO₂ due to the acidic environment. During the phase transformation in step III, the inner core of the γ-MnO₂ microsphere dissolves and then recrystallizes to form β-MnO₂ nanorods on the outer surface, creating an internal cavity. Finally, in step IV, the cavity size and microsphere diameter are enlarged through the dissolution-crystallization process. The formation of hollow β-MnO₂ microspheres was characterized by SEM (Fig. 5e).

**Fig. 6:**
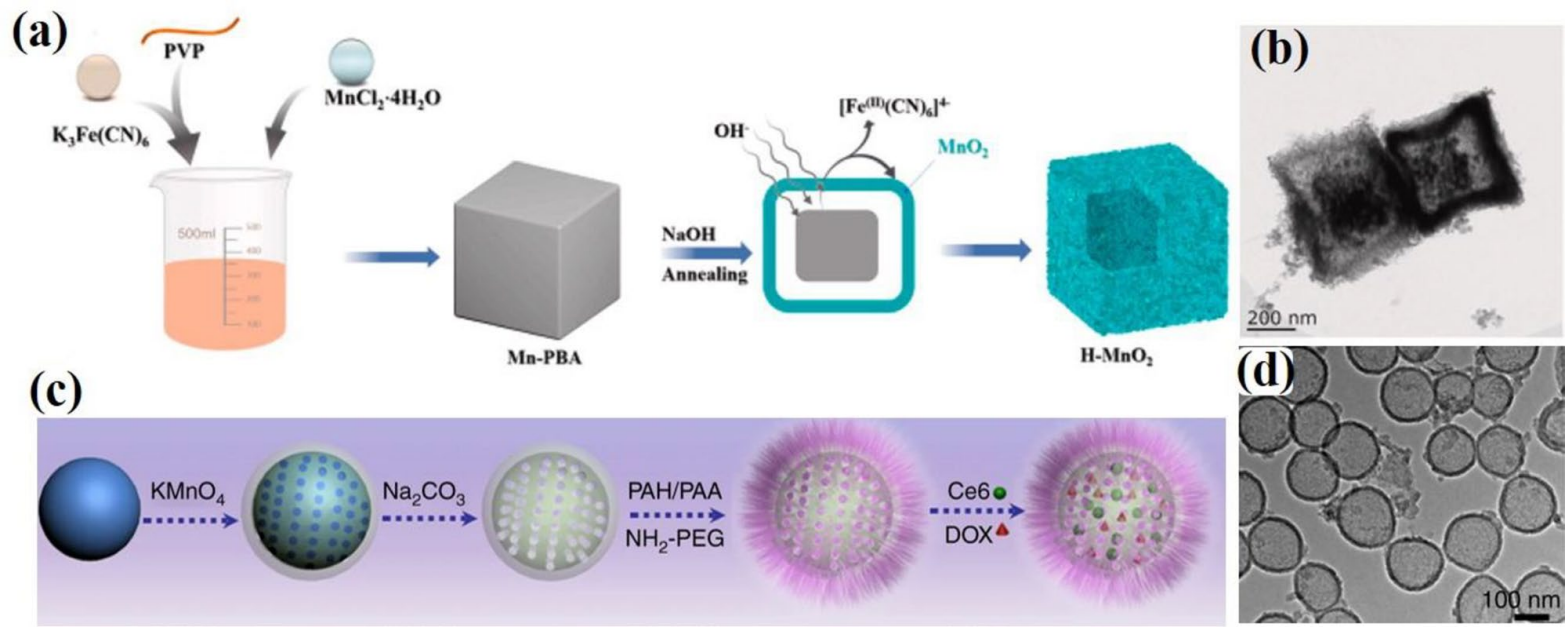
Schematic illustration of the synthesis of H-MnO₂ nanostructures: (**a**) Synthesis process of H–MnO₂ nanozymes *via* a self-template sacrifice and redox strategy. Reproduced with permission [75]. (**b**) TEM image of H-MnO₂. (**c)** Synthesis process of H-MnO₂-PEG nanoshells *via* a hard templating method; (**d**) TEM image of H-MnO₂ nanoshells. Reproduced with permission [46]

**Fig. 7:**
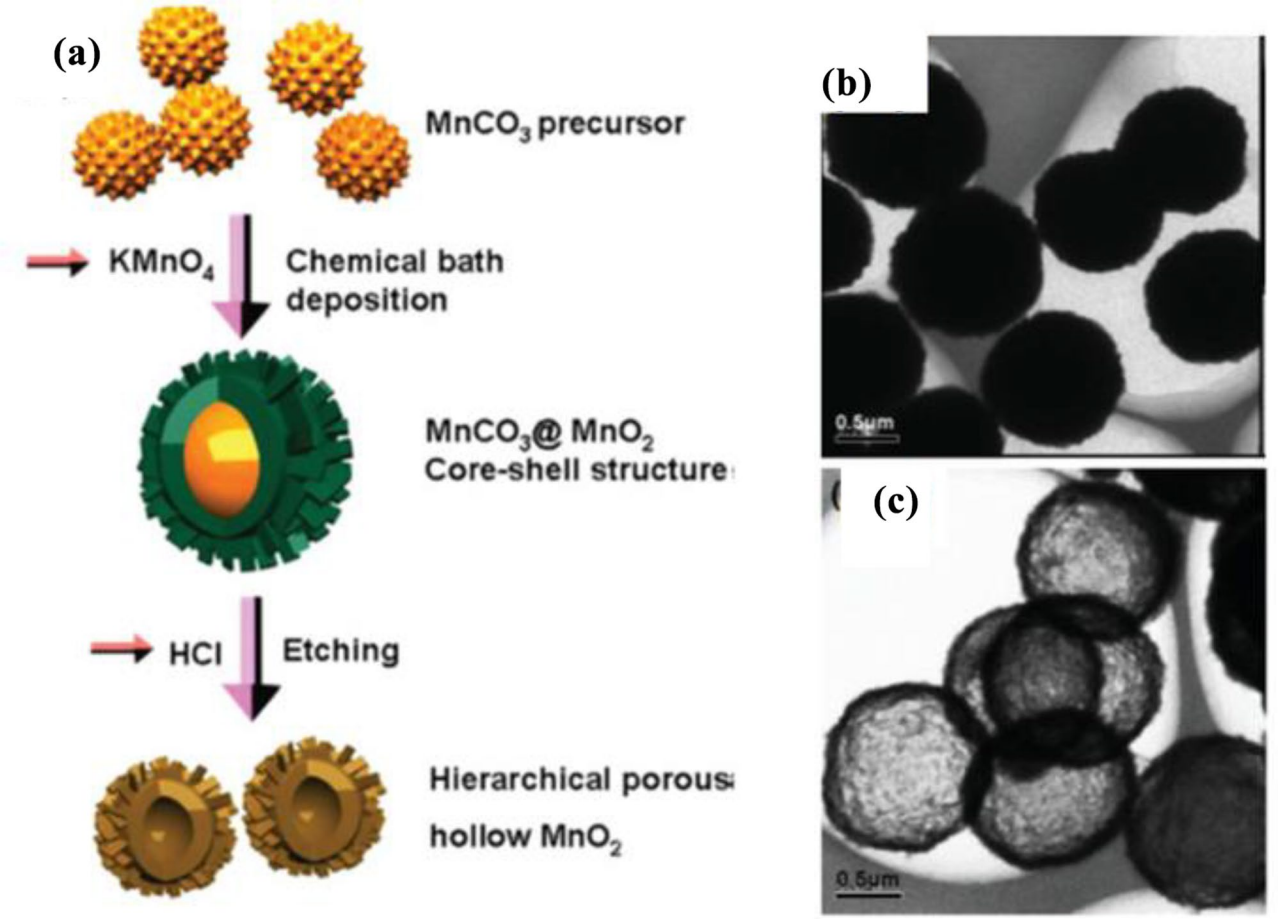
(**a**) Schematic illustration of the facile synthesis of hierarchical hollow MnO₂ spheres. (**b**) FESEM image of MnCO₃ sphere used as a precursor. (**c**) TEM image of ε-MnO₂ hollow sphere. Reproduced with permission [79]

Similarly, Shu and co-workers developed triethanolamine-induced hollow MnO₂ nanoboxes (H-MnO₂-TEA) using a self-templated process [72]. Ma et al. also successfully prepared birnessite-type MnO₂ hollow structures through a self-template route by directly reacting an aqueous solution of KMnO₄ with solid MnCO₃ precursor crystals, followed by the removal of the MnCO₃ core using HCl [73]. This process involved three steps: first, MnCO₃ precursors were synthesized by reacting an aqueous solution of MnSO₄ with NaHCO₃. The surfaces of the prepared MnCO₃ were then oxidized by KMnO₄ to form a MnO₂ core–shell structure. Finally, the MnCO₃ core was removed with an HCl aqueous solution, resulting in the formation of MnO₂ hollow structures. In 2017, Shu et al. developed a method for synthesizing 3D porous hollow MnO microspheres (PHMSs) using a self-template approach [74]. As illustrated in Fig. 5f, Mn(CH₃COO)₂·4 H₂O and Na₂CO₃ were dissolved in distilled water to create two homogeneous solutions. The Na₂CO₃ solution was added to the Mn(CH₃COO)₂·4 H₂O solution under constant stirring, and the mixture was stirred at room temperature for 1 h to form the MnCO₃ precursor. The surface of the MnCO₃ precursor was then oxidized with KMnO₄ solution at room temperature, yielding the MnCO₃@δ-MnO₂ core/shell structure. Finally, 3D δ-MnO₂ PHMSs were obtained by removing the MnCO₃ core with HCl, and further calcination of the δ-MnO₂ product at 400 °C for 4 h produced 3D α-MnO₂ PHMSs. The obtained α-MnO_2_ PHMSs were characterized by SEM (Fig. 5g). Recently, Yue et al. developed a novel method for synthesizing H–MnO₂ nanozymes using a self-template sacrifice and redox strategy, unlocking new possibilities for the efficient production of hollow MnO₂-based nanozymes with promising applications in catalysis and biomedicine [75]. The synthetic process, depicted in Fig. 6a, begins with the preparation of Mn-PBA precursors by dissolving K₃Fe(CN)₆·3 H₂O and PVP (K30) in deionized water. A solution of MnCl₂·4 H₂O was then prepared separately and added to the reaction mixture under stirring, resulting in a gradual color change from yellow to brown. After stirring for 5 min, the solution was allowed to stand at 25 °C in the dark for 24 h. The precipitate was collected by centrifugation, washed with deionized water and ethanol, and dried under vacuum at 60 °C to obtain Mn-PBA. Subsequently, H–MnO₂ cubes were synthesized by dissolving Mn-PBA in ethanol and sonicating for 3 min. Then, a 0.1 M NaOH solution was added, followed by thorough mixing and heating at 40 °C for 1 h. The resulting compound was washed multiple times via centrifugation with deionized water and ethanol. Finally, H–MnO₂ was obtained by drying at 60 °C for 12 h and characterized by TEM (Fig. 6b). In contrast, the successful coating or deposition of the target material onto the template, followed by an effective template removal process, is crucial for the synthesis of hard templates [76, 77]. This method relies on the precise fabrication of a template to ensure that the resulting nanostructures maintain their desired shapes and morphologies after the template is removed. The proper execution of these steps is essential for achieving high-quality nanomaterials with specific functional properties. In hard templating synthesis approach, a solid material (often silica or polymer spheres) serves as a hard template around which the MnO₂ layer is deposited. After the MnO₂ shell is formed, the core template is removed through chemical etching or dissolution, resulting in a hollow structure. Although this method allows precise control of the size and shape of the hollow nanostructure, it often involves multiple steps and the use of chemicals to remove the hard template. In 2004, Sasaki et al. prepared ultrathin hollow nanoshells of Mn₂O₃ through a layer-by-layer assembly method involving exfoliated MnO₂ nanosheets and polyelectrolytes on polymer bead templates, followed by removal of the polymer cores *via* calcination [78]. This process yielded well-defined hollow spherical nanoshells with diameters of 350–380 nm, and ultrathin shell walls with diameters of 10–15 nm. This method utilizes polyethylenimine (PEI), a positively charged polyelectrolyte, as a binder, and poly(methyl methacrylate) (PMMA) spheres (400 nm in diameter) as templates. Initially, the PMMA beads were ultrasonically dispersed in water containing PEI to precoat their surfaces, followed by centrifugation and washing to remove excess PEI. The MnO₂ nanosheets were then adsorbed onto the PEI-coated PMMA beads by adding a colloidal suspension of MnO₂ dropwise under constant stirring, and the resulting products were recovered through centrifugation and washing. Multilayered core–shell composites, (PEI/MnO_2_)_n_, were prepared by repeating the coating process multiple times. Finally, calcination at 450 °C in air produced hollow Mn₂O₃ nanoshells. Hierarchical hollow ε-MnO₂ spheres were synthesized by Liu et al. using MnCO₃ spheres as reactive templates, which were reacted with KMnO₄, followed by the selective removal of manganese carbonate using HCl [79]. The stepwise process for obtaining these hollow structures is illustrated in Fig. 7a. Initially, MnCO₃ spheres were synthesized by mixing aqueous solutions of MnSO₄ and NaHCO₃ at room temperature for 3 h. The MnCO₃ spheres were filtered, washed with distilled water, air-dried at 60 °C for 12 h, and characterized by FESEM (Fig. 7b). Subsequently, the MnCO₃ spheres were dispersed in distilled water, and KMnO₄ solution was added with constant stirring for 2 min. HCl (2.5 M) was added, and the mixture was stirred for 1 min at room temperature. Finally, the hollow ε-MnO₂ spheres were separated, washed with double-distilled water, vacuum-dried at 60 °C for 12 h, and characterized by TEM (Fig. 7c). Liu et al. pioneered a novel methodology for synthesizing PEG-coated hollow mesoporous MnO₂ (H-MnO₂-PEG) nanoshells, utilizing monodispersed sSiO₂ NPs as a hard template [46]. A schematic of the synthesis process is shown in Fig. 6c. Initially, monodisperse sSiO₂ NPs were synthesized, which involves the hydrolysis of tetraethyl orthosilicate (TEOS) in a water and alcohol mixture in the presence of ammonia solution. A uniform layer of mesoporous MnO₂ was then grown on the surface of the SiO₂ NPs by dropwise adding an aqueous solution of KMnO₄ into the sSiO₂ NP suspension under ultrasonication for 6 h through a redox reaction. The resulting MnO₂-coated SiO₂ (SiO₂@MnO₂) NPs were centrifuged at 14,800 rpm and washed several times with distilled water. To obtain the hollow structure, the sSiO₂ cores were dissolved in 2 M Na₂CO₃ solution and heated at 60 °C for 12 h. Finally, the H-MnO₂ nanoshells were separated via centrifugation and washed thoroughly with water. The obtained H-MnO₂ nanoshells were characterized using TEM (Fig. 6d).

**Fig. 8:**
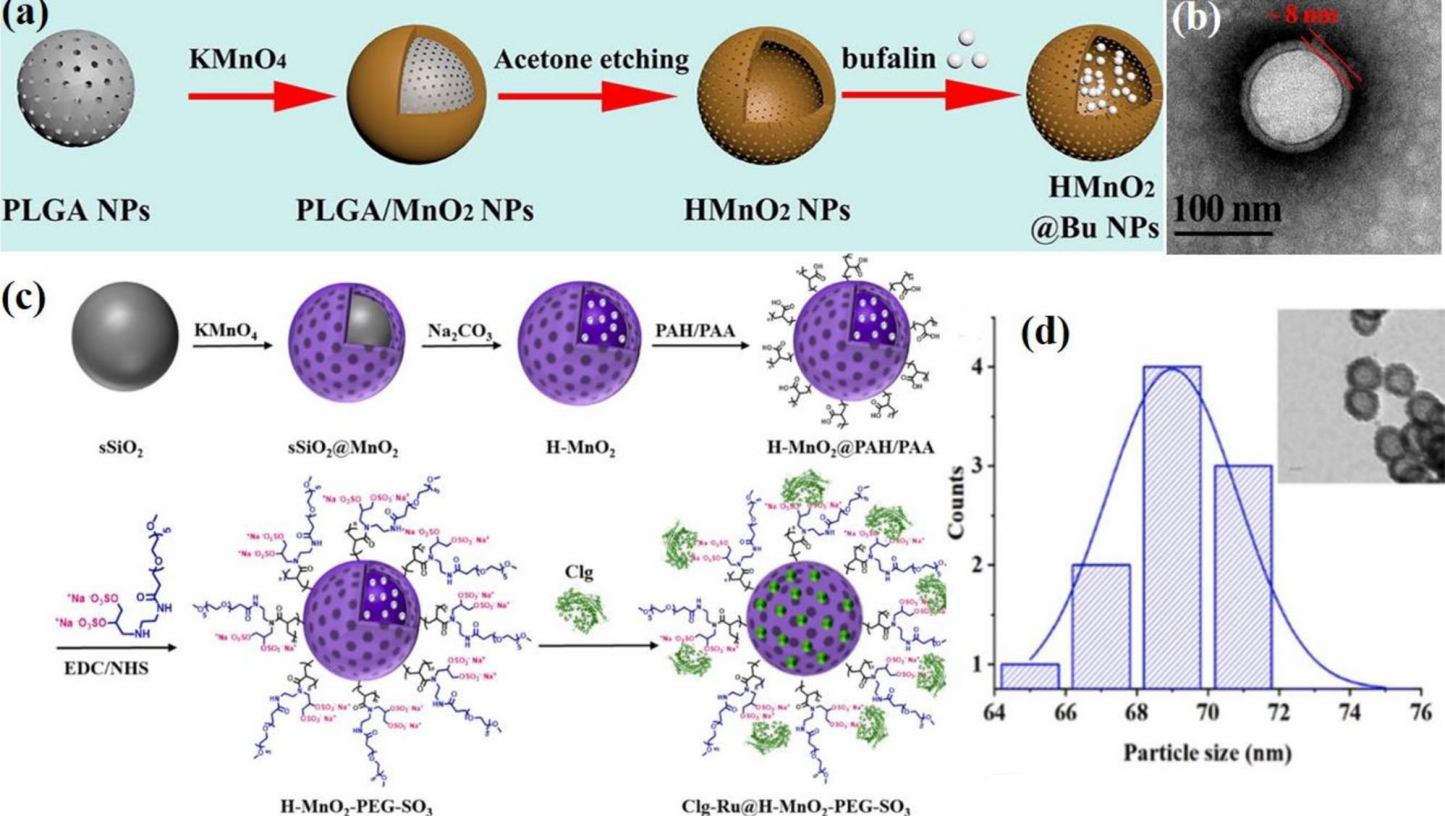
Schematic illustration depicting the step-by-step synthesis process for H-MnO₂ nanocomposites. (**a**) Preparation process of H-MnO₂@Bu NPs, using poly(lactic-co-glycolic acid) (PLGA) as a hard template. (**b**) TEM image of H-MnO_2_ NPs. Reproduced with permission [83]. (**c**) Synthesis process for Clg-Ru@H–MnO₂–PEG–SO₃ using solid silica (sSiO₂) as a hard template. (**d**) TEM image and particle size distribution of H–MnO_2_–PEG–SO_3_ NPs Reproduced with permission [80]

To enhance the biocompatibility of the H–MnO₂-based drug delivery system, our group recently introduced an effective method for synthesizing mesoporous Clg@H–MnO₂–PEG–SO₃ NPs [80]. A schematic illustration of this process is shown in Fig. 8c. The novel bifunctional design of –SO₃–PEG-amine endows H–MnO₂–PEG–SO₃ NPs with multiple advantages, including improved water solubility, enhanced physiological stability, prolonged circulation time, and increased tumor accumulation through enhanced permeation and retention (EPR) effect. This design also facilitated intracellular drug delivery. Additionally, the sulfate group (–SO₃) on the PEGylated amine enables the trapping of the protease enzyme collagenase through electrostatic interactions, further enhancing the therapeutic potential of the system. The process involves several key steps such as (a) Template Preparation: Monodisperse SiO₂ NPs were first synthesized as templates *via* the hydrolysis of TEOS. Solid SiO₂ (sSiO₂) NPs were prepared using an inverse microemulsion method. Triton X-100, cyclohexane, and n-hexanol were uniformly stirred at room temperature for 5 min. Water and ammonia were then added dropwise and the mixture was stirred for 30 min. Subsequently, (3-aminopropyl)triethoxysilane (APTES) and TEOS were slowly added to the flask, and the reaction was allowed to continue for 24 h at 25 °C. The sSiO₂ NPs were then collected by centrifugation (14,800 rpm, 10 min) and washed three times with ethanol and water. (b) Coating with MnO₂: An aqueous solution of KMnO₄ was added dropwise to a suspension of sSiO₂ NPs under ultrasonication. The reaction was allowed to proceed under stirring for 8 h. The mesoporous sSiO₂@MnO₂ NPs were collected by centrifugation at 14,800 rpm for 10 min. (c) Formation of hollow structures: The sSiO₂@MnO₂ NPs were dispersed in a Na₂CO₃ solution and stirred at 60 °C for 12 h to dissolve the internal silica. Mesoporous H–MnO₂ NPs were obtained through centrifugation at 14,800 rpm, followed by washing with water three times. To functionalize the H–MnO₂ NPs, a layer-by-layer assembly process was employed. First, the cationic polymer poly(allylamine hydrochloride) (PAH) and the anionic polymer poly(acrylic acid) (PAA) were successively coated onto the negatively charged surface of the H–MnO₂ NPs through electrostatic interactions. Following this, PEGylated amine (–SO₃–PEG-amine) was conjugated to the carboxyl groups on the PAA-coated H–MnO₂ NPs *via* amide coupling, thus forming H–MnO₂–PEG–SO₃ NPs. Finally, Clg@H–MnO₂–PEG–SO₃ was prepared by the dropwise addition of an aqueous enzyme solution in phosphate buffer (PB) to H–MnO₂–PEG–SO₃. The particle size of MnO₂–PEG–SO₃ NPs was determined using TEM (Fig. 8d).

Recently, Li et al. designed and synthesized novel intelligent H-MnO₂ NPs with functional genes, employing a similar synthetic strategy to develop them as nanovaccines [12]. Recently, these synthetic methods have gained widespread use in research, demonstrating their remarkable reproducibility and practical applicability [11, 14, 81]. As research in this field has advanced significantly, numerous new templates for synthesizing H-MnO₂ NPs have been developed. Zhu et al. devised an excellent method for synthesizing H-MnO₂ NPs using poly(lactic-co-glycolic acid) (PLGA) as a hard template, as illustrated in Fig. 8a [82, 83]. Initially, porous PLGA NPs were prepared using a nanoprecipitation method, which was then utilized as templates for the growth of the MnO₂ shell. This growth resulted from the redox reaction between hydroxyl groups on the surface of the PLGA NPs and excess KMnO₄, yielding PLGA@MnO₂ NPs. Subsequently, the PLGA cores were removed *via* acetone etching to produce H-MnO₂ NPs, which were then employed to deliver bufalin, a potent small molecule inhibitor. In this process, PLGA and TPGS (D-α-Tocopherol polyethylene glycol succinate, a pore-forming agent) were first dissolved in acetone. The resultant solution was then added dropwise to deionized water while stirring at 1000 rpm for 3 h, after which the residual acetone was evaporated at 25 °C in vacuo. The obtained PLGA dispersion was centrifuged and washed three times with deionized water to allow TPGS to leach out, thus creating porous nanoparticles. Next, an aqueous solution of KMnO₄ was added dropwise to the PLGA solution under constant stirring at 25 °C for 24 h. The PLGA cores were then removed by dispersing the NPs in acetone. Finally, the sample was washed three times with deionized water and freeze-dried for 48 h to obtain the HMnO₂ NPs. TEM characterization revealed a hollow structure with an outer layer thickness of approximately 8 nm (Fig. 8b). Importantly, hard-templating methods offer significant advantages for preparing highly uniform hollow nanostructures, including highly tunable particle sizes, controllable morphologies, and excellent monodispersity. However, these methods have limitations, particularly in synthesizing non-spherical hollow structures, owing to the scarcity of suitable templates. Despite being widely regarded as the most popular approach for fabricating hollow nanostructures, hard-templating methods typically involve multiple steps and require additional surface-modification procedures to achieve heterogeneous coatings. These complexities reduce synthesis efficiency and lead to higher production costs.

### Chemical properties of MnO_2_-based nanomaterial

It is well established that the TME is characterized by hypoxia, mild acidity, and excessive production of GSH and H₂O₂ [16]. To overcome these barriers effectively, MnO_2_-based nanomaterials are often engineered to modulate and adjust the TME, thereby enhancing therapeutic outcomes by improving drug delivery, reducing resistance mechanisms, and creating more favorable conditions for cancer treatment. In this section, we summarize the chemical properties of MnO₂, focusing on its interactions with TME [84]. Notably, these manganese oxides exhibit acid-responsive behaviors and catalase-like activity, catalyzing the conversion of H⁺/H₂O₂ into oxygen (O₂) and Mn²⁺.

$$\:\text{M}\text{n}{\text{O}}_{2}+{\text{H}}_{2}{\text{O}}_{2}+2{\text{H}}^{+}\to\:\:{\text{M}\text{n}}^{2+}+2{\text{H}}_{2}\text{O}+{\text{O}}_{2}$$

In addition, these oxidizing MONs can mimic glutathione peroxidase (GPx) by oxidizing GSH to GSSH [85].

$$\:\text{M}\text{n}{\text{O}}_{2}+2\text{G}\text{S}\text{H}+2{\text{H}}^{+}\to\:\:{\text{M}\text{n}}^{2+}+\text{G}\text{S}\text{S}\text{H}+{2\text{H}}_{2}\text{O}$$

The Mn²⁺ generated can further catalyze H₂O₂, producing hydroxyl radicals (•OH) and Mn³⁺ through Fenton-like reactions [86].

$$\:{\text{M}\text{n}}^{2+}+{\text{H}}_{2}{\text{O}}_{2}\:\to\:\:{\text{M}\text{n}}^{3+}+\:{\bullet\:}\text{O}\text{H}+\text{O}{\text{H}}^{-}$$

The Mn³⁺ then catalyzes H₂O₂ to form superoxide anions (•O₂⁻) and regenerates Mn²⁺

$$\:{\text{M}\text{n}}^{3+}+{\text{H}}_{2}{\text{O}}_{2}+\text{O}{\text{H}}^{-}\:\to\:\:{\text{M}\text{n}}^{2+}+\:{\bullet\:}{\text{O}}_{2}-\:+2{\text{H}}_{2}\text{O}$$

These reactions demonstrate that MONs are not only ideal TME-responsive materials but also actively modulate the TME by increasing pH, depleting H₂O₂ and GSH, raising oxygen levels, and generating dissociative Mn²⁺ ions [87]. Owing to their unique and remarkable properties, MONs are increasingly being explored for precise drug and gene delivery as well as for enhancing therapeutic strategies.

### Toxicity

Before employing nanomaterials, it is crucial to evaluate their biological toxicity. Thorough assessments must ensure that the material exhibits low toxicity both in vivo and in vitro before advancing to clinical trials [88]. Notably, the H-MnO_2_ NPs demonstrated high cell viability even at elevated concentrations, indicating low cytotoxicity and excellent biocompatibility [37]. The results from in vivo studies reveal that Mn²⁺ released from the decomposition of H-MnO_2_ NPs are efficiently cleared by the kidneys [46]. Additionally, Liu et al. in their study substantiated the low tissue toxicity and high biocompatibility of H-MnO_2_ NPs, as evidenced by hematoxylin and eosin (H&E) staining of vital organs, including the heart, liver, spleen, lungs, and kidneys. In a recent study by Shi et al., H-MnO_2_ NPs were intravenously administered to healthy female Kunming mice [89]. A comprehensive analysis of various blood parameters, including red blood cell count (RBC), white blood cell count (WBC), mean corpuscular hemoglobin concentration (MCHC), hemoglobin (HGB), aspartate aminotransferase (AST), alanine aminotransferase (ALT), alkaline phosphatase (ALP), and hematocrit (HCT) levels, showed no significant differences when compared to the control group. These findings further underscore the exceptional biocompatibility of H-MnO_2_ NPs, indicating their potential for safe applications in the biomedical field.

However, some past studies suggested that H-MnO_2_ NPs are toxic to both healthy and cancerous cells [90]. This toxicity is believed to arise from the accumulation of H-MnO_2_ NPs which appears to be more detrimental to cancer cells than to healthy ones, independent of whether the MnO₂ is modified with dopamine. Moreover, degraded H-MnO₂ in conjunction with antioxidants has shown a beneficial impact on healthy cells while adversely affecting cancer cells in the initial treatment stages. The rapid dissolution rates of H-MnO_2_ NPs also indicated their excellent biodegradability, making them promising candidates for targeted drug delivery in cancer therapy.

## Cancer treatment

### TME-responsive drug release

Because cancer cells require high ATP production for frequent proliferation, they produce more ROSs (especially H_2_O_2_) and protons than normal cells. Accordingly, the TME was weakly acidic at pH 6.3. MnO_2_ nanocarriers release encapsulated anticancer drugs as they decompose into Mn^2+^ in the TME, and simultaneously O_2_ is released through the following TME-responsive reaction:

$$\:\text{M}\text{n}{\text{O}}_{2}+2{\text{H}}_{2}{\text{O}}_{2}+2{\text{H}}^{+}={\text{M}\text{n}}^{2+}+4{\text{H}}_{2}\text{O}+{\text{O}}_{2}$$

In addition, because Mn^2+^ has paramagnetic properties, it can act as an MRI probe. The types of drugs transported by MnO_2_ nanocarriers include chemodrugs, photosensitizers, and siRNAs. MnO_2_ can play the role of a magnificent TME-responsive anticancer drug carrier.

**Fig. 9:**
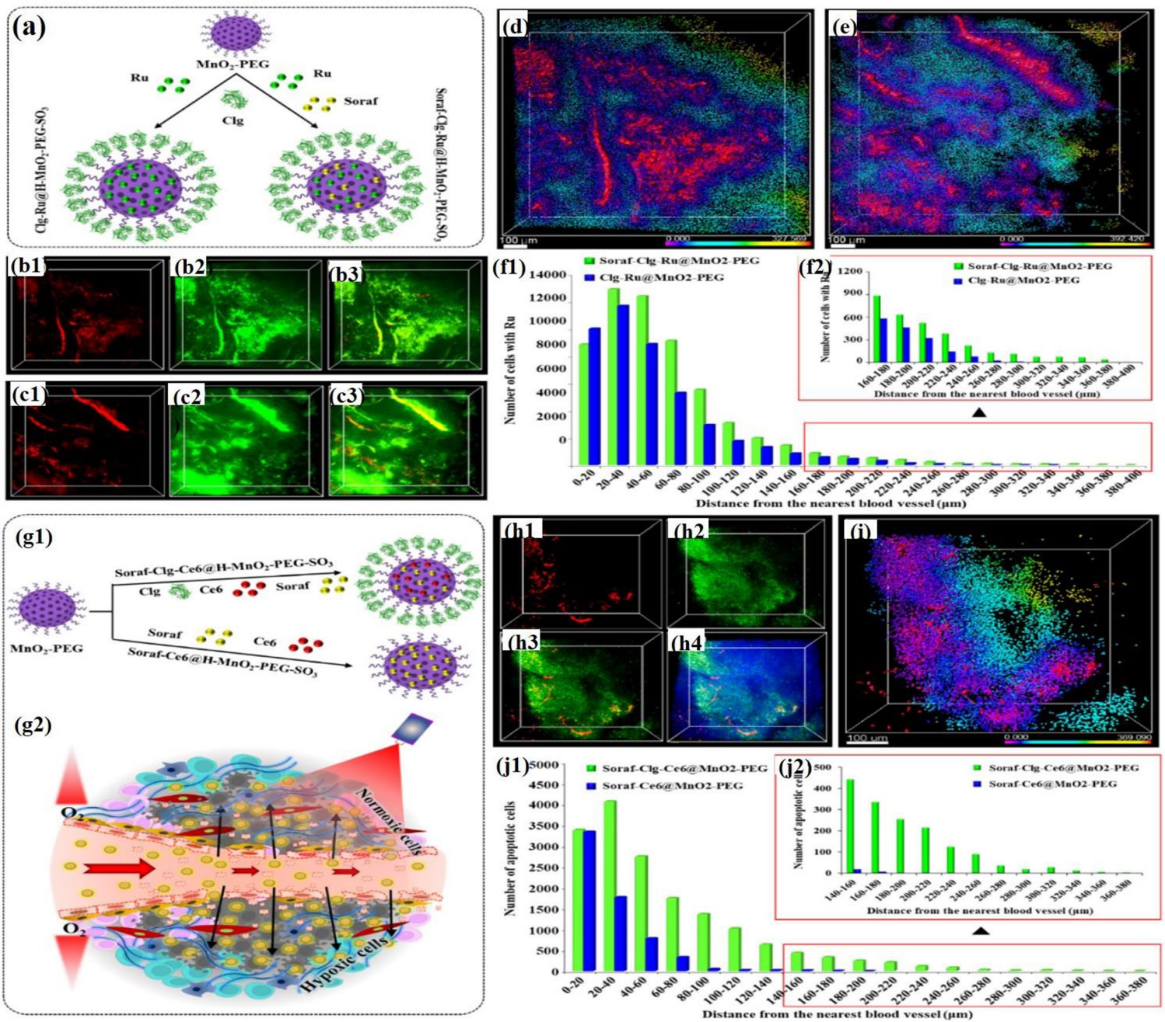
(**a–f**) Comparison of Ru penetration with collagenase and antiangiogenic agent. (**a**) Schematic of Clg-Ru@H–MnO₂–PEG–SO₃ and Soraf-Clg-Ru@H–MnO₂–PEG–SO₃ preparation. (**b1-b3**) 3D images of Ru in tumor tissues after Clg-Ru@H–MnO₂–PEG–SO₃ injection. (**c1-c3**) 3D images after Soraf-Clg-Ru@H–MnO₂–PEG–SO₃ injection. (**d-e**) 3D reconstructions showing Ru penetration distribution. (**f1-f2**) Graphs showing Ru distribution based on distance from blood vessels. (**g-j**) Comparison of apoptosis induced by Soraf-Clg-Ce6@H–MnO₂–PEG–SO₃ vs. SorafCe6@H–MnO₂–PEG–SO₃, with 3D images and graphs showing apoptotic cell distribution. Reproduced with permission [80]

As aforementioned, our group developed an innovative bioenzyme-conjugated nanodrug delivery system with enhanced drug permeability to eliminate hypoxic cancer cells within the TME [80]. A key challenge in the treatment of tumors is the dense extracellular matrix (ECM), which acts as a physical barrier that hinders drug penetration. In this study, a nanoformulation was designed to specifically target the collagen component of ECM by locally delivering collagenase type-I. The resulting formulation, Clg@H–MnO_2_–PEG–SO_3_, effectively degraded collagen in 4T1 tumors. This was confirmed by the enhanced release of ruthenium (Ru) within the TME, highlighting its potential to improve drug penetration and efficacy in hypoxic tumors. Figure 9a illustrates the schematic for the preparation of Clg-Ru@H–MnO₂–PEG–SO₃ NPs and Soraf-Clg-Ru@H–MnO₂–PEG–SO₃ NPs. Interestingly, as depicted in Fig. 9b3, the merged images of the blood vessels and Ru distribution revealed significant Ru release within the TME. The extent of Ru released from the blood vessels was so substantial that it obscured the vascular structure. This image highlights the remarkable efficacy of Clg-Ru@H–MnO₂–PEG–SO₃ NPs in enhancing drug delivery. The 3D-reconstructed image (Fig. 9d) demonstrates that the distribution of ruthenium (Ru) from Clg-Ru@H–MnO₂–PEG–SO₃ extended up to 327 μm from the blood vessels (DPDmax). The quantity of Ru released from Clg-Ru@H–MnO₂–PEG–SO₃ was significantly higher than that released from Soraf-Ru@H–MnO₂–PEG, underscoring the superior drug delivery capabilities of the Clg-Ru formulation. This enhanced release suggests more effective penetration of the TME, leading to improved therapeutic outcomes. Compared to the antiangiogenic effect on drug delivery, the inclusion of the ECM-degrading bioenzyme collagenase resulted in a remarkable exponential increase in DPDmax, extending it by 171 μm. The enhanced drug delivery effect resulting from the combination of antiangiogenic drugs and the ECM-degrading bioenzyme collagenase was further examined in tumor tissues treated with Soraf-Clg-Ru@H–MnO₂–PEG–SO₃ (Fig. 9c1–c3). The analysis of the 3D-reconstructed image (Fig. 9e) revealed that the distribution of Ru from Soraf-Clg-Ru@H–MnO₂–PEG–SO₃ extended up to 392 μm DPDmax from the blood vessels. In contrast, the bioenzyme alone achieved a DPDmax of 327 μm (Fig. 9d). The combination of the antiangiogenic agent with collagenase resulted in a significant increase of 65 μm in the DPDmax (Fig. 9e), highlighting the enhanced drug delivery capability of this synergistic approach. Additionally, the total amount of Ru released from Soraf-Clg-Ru@H–MnO₂–PEG–SO₃ was found to be slightly higher than that from Clg-Ru@H–MnO₂–PEG–SO₃, attributed to the synergistic effect of the combination (Fig. 9f1 and f2). These results demonstrate that utilizing the ECM-degrading bioenzyme collagenase effectively enhances drug delivery to the hypoxic regions of the TME compared to alternative drug delivery strategies. Finally, the distribution of apoptotic cell death induced by Soraf-Clg-Ce6@H–MnO_2_–PEG–SO_3_ was compared with that of Soraf-Ce6@H–MnO_2_–PEG–SO_3_ within the TME (Fig. 9g1 and 9g2). The ECM-degrading action of collagenase when combined with sorafenib and PDT (Fig. 9h3), resulted in a more widespread distribution of apoptotic cells compared to the combination of sorafenib and PDT alone. The 3D distribution of apoptotic expression, represented by spectral spots, ranged from 0 to 369 μm CDDmax (Fig. 9i). The spatial distribution of apoptotic cells induced by Soraf-Clg-Ce6@H–MnO₂–PEG–SO₃, which combines the ECM-degrading bioenzyme, antiangiogenic agent, and PDT effect, was significantly greater than that induced by Soraf-Ce6@H–MnO₂–PEG–SO₃, which lacks the collagenase effect (Fig. 9j1 and j2). These results demonstrate that H–MnO₂–PEG–SO₃ loaded with collagenase significantly increases the extent of apoptosis compared to other treatment modalities lacking collagenase, primarily by enhancing drug penetration into the TME.

**Fig. 10:**
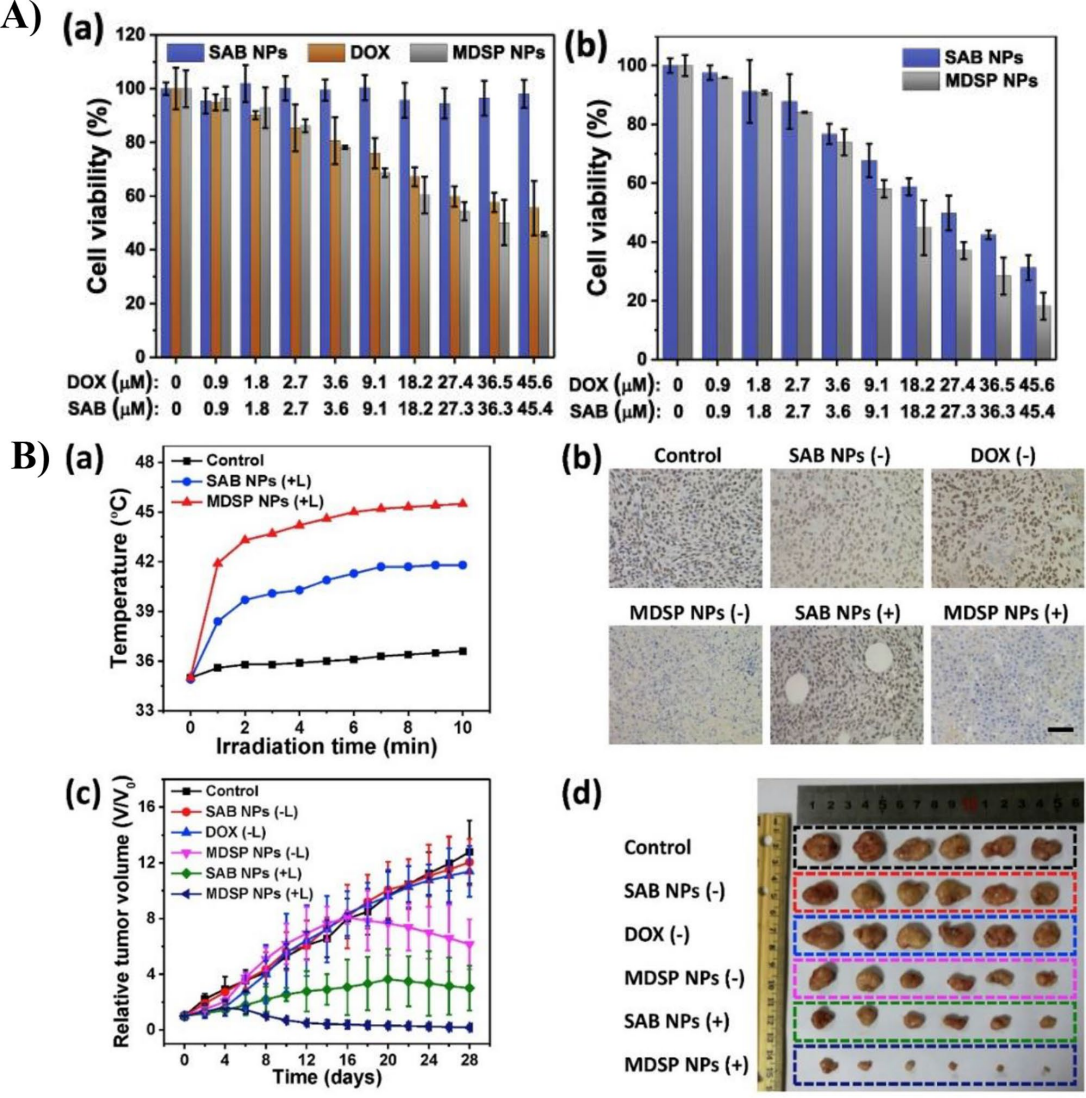
(**A**) The in vitro cell viability test of HCT-116 cells. (**a**) Cell viability of HCT-116 after different treatments without xenon laser irradiation. (**b**) Cell viability of HCT-116 after different NP treatments with xenon laser irradiation. **B**) The in vivo test of tumor bearing mice. (**a**) Temperature of tumor under irradiation after i.v. injection of saline and NPs. (**b**) Immunostaining images of HIF-1α in tumor tissue after different treatments. (**c**) Relative tumor volume according to different injection groups of mice. (**d**) Comparison of size of tumor extirpated from mice of different injection groups. Reproduced with permission [91]

Tang et al. established a TME-responsive degradable nanoplatform (MDSP NP) by loading the chemotherapeutic drug doxorubicin and aza-BODIPY photosensitizer (SAB) onto MnO_2_ nanocarriers for the treatment of cancer cells in hypoxic regions [91]. In addition, the MDSP NPs showed strong near-infrared absorption (~ 853 nm), allowing them to exhibit photothermal activity (Fig. 10A). shows the viability of the HCT-116 cells after treatment with MDSP, SAB, or DOX NPs. Compared to SAB NPs and DOX, MDSP NPs had the greatest anticancer effect, and the therapeutic effects of NPs were maximized when xenon laser irradiation was used. Figure 10B shows the results of in vivo experiments treating tumor-bearing mice with i.v. injections of MDSP NP, SAB NP, and DOX. Consequently, the MDSP NPs with laser irradiation had the greatest effect on the regulation of HIF-1α and tumor size reduction. Synergistic chemo/photodynamic/photothermal therapy with MnO_2_ has great potential for overcoming hypoxia and enhancing the therapeutic effects.

### Circumventing Multidrug resistance (MDR)

**Fig. 11:**
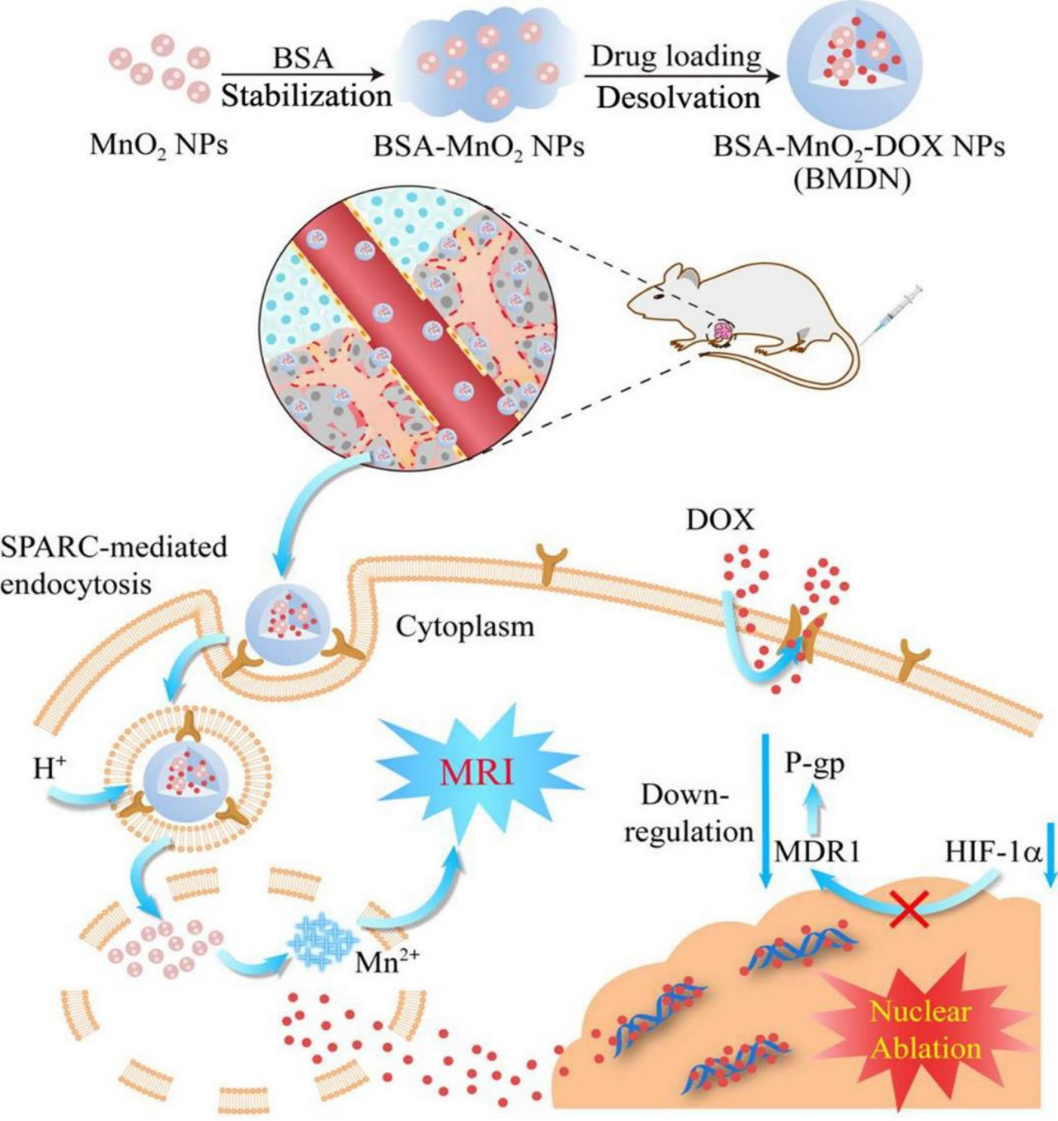
Schematic diagrams of assembly pathway of BSA-MnO₂-DOX nanoparticles and pathway of drug release and the inhibition of multidrug resistance (MDR). Reproduced with permission [92]

MDR is a mechanism that releases various types of anticancer drugs from cells. This is a major obstacle to chemotherapy. Hypoxia leads to the production of hypoxia-inducible factor-1a (HIF-1α). HIF-1α regulates expressions of various hypoxia-response elements, including multidrug resistance gene 1 (MDR1). However, HIF-1α is unstable under oxygen-rich conditions. Because MnO_2_ can downregulate the expression of MDR1 by generating O_2_ in the TME environment, it has received great attention not only as a nanocarrier but also as a treatment to circumvent MDR. Du et al. loaded the anticancer drug DOX into BSA-MnO_2_ NPs to overcome MDR (Fig. 11) [92]. In both in vitro and in vivo experiments, BSA-MnO_2_-DOX NPs significantly reduced cancer cell viability, hypoxia-related protein expression, and tumor size compared with free DOX and other NPs.

### MnO₂-mediated immunotherapy

Immunotherapy is one of the most promising strategies for combating tumors because it stimulates the immune system and enhances its innate capacity to identify and attack cancer cells. TME-responsive MnO₂-based strategies have many advantages to overcome TME. One of the important things is O₂ generation because it can attenuate tumor hypoxia. Hypoxia is a characteristic feature of solid tumors and is induced by an imbalance between the high oxygen consumption of rapidly proliferating cancer cells and the low oxygen supply from the abnormal vasculature. Hypoxia not only promotes a tumor-progressive environment but also induces severe therapeutic resistance in chemo-, radio-, and immunotherapies, which require sufficient oxygen for maximum efficacy. Tumor hypoxia contributes to macrophage recruitment and promotes macrophage polarization to M2-like tumor-associated macrophages (TAMs), leading to immunosuppression. TAMs in hypoxic regions play an important role in promoting tumor progression through various mechanisms, including secretion of anti-inflammatory cytokines, promotion of angiogenesis and metastasis, ECM remodeling, stimulation of cancer cell proliferation, and inhibition of anti-tumor immune cells. Since TAMs constitute approximately 50% of the tumor mass and still have the plasticity to convert into the M1 phenotype, re-educating M2-like TAMs into the M1 phenotype to suppress their tumor-promoting functions and activate immunological activities could be an effective therapeutic strategy. Recently, the Song group utilized MnO₂ NPs to simultaneously attenuate tumor hypoxia and relieve acidic pH. Because MnO₂ reacts with H₂O₂ and H⁺, it results in an increased oxygen level and a decreased hydrogen ion concentration within the tumor microenvironment. Furthermore, they modified MnO₂ NPs with mannans (Man) and hyaluronic acid (HA) to re-polarize M2 TAMs into pro-inflammatory, anti-tumoral M1 macrophages. Re-polarized M1 macrophages activated by immunogenic HA secrete H₂O₂ via their innate inflammatory response pathways, which further enhances the reaction of MnO₂ NPs with H₂O₂. Mice injected with Man-HA-MnO₂ NPs showed an increase in iNOS⁺ M1 macrophages and the pro-inflammatory cytokine IL-12, along with a decrease in CD206⁺ M2 macrophages and the anti-inflammatory cytokine IL-10, indicating the re-polarization of M2 TAMs to anti-tumoral M1 macrophages. Additionally, a strikingly decreased tumor hypoxic region was observed via tissue immunofluorescence staining with hypoxia markers such as anti-Pimonidazole, HIF-1α, and VEGF antibodies. All of these features indicate that Man-HA-MnO₂ NPs successfully accumulated in the tumor and subsequently modulated the tumor microenvironment into an immune-favorable condition [18].

**Fig. 12:**
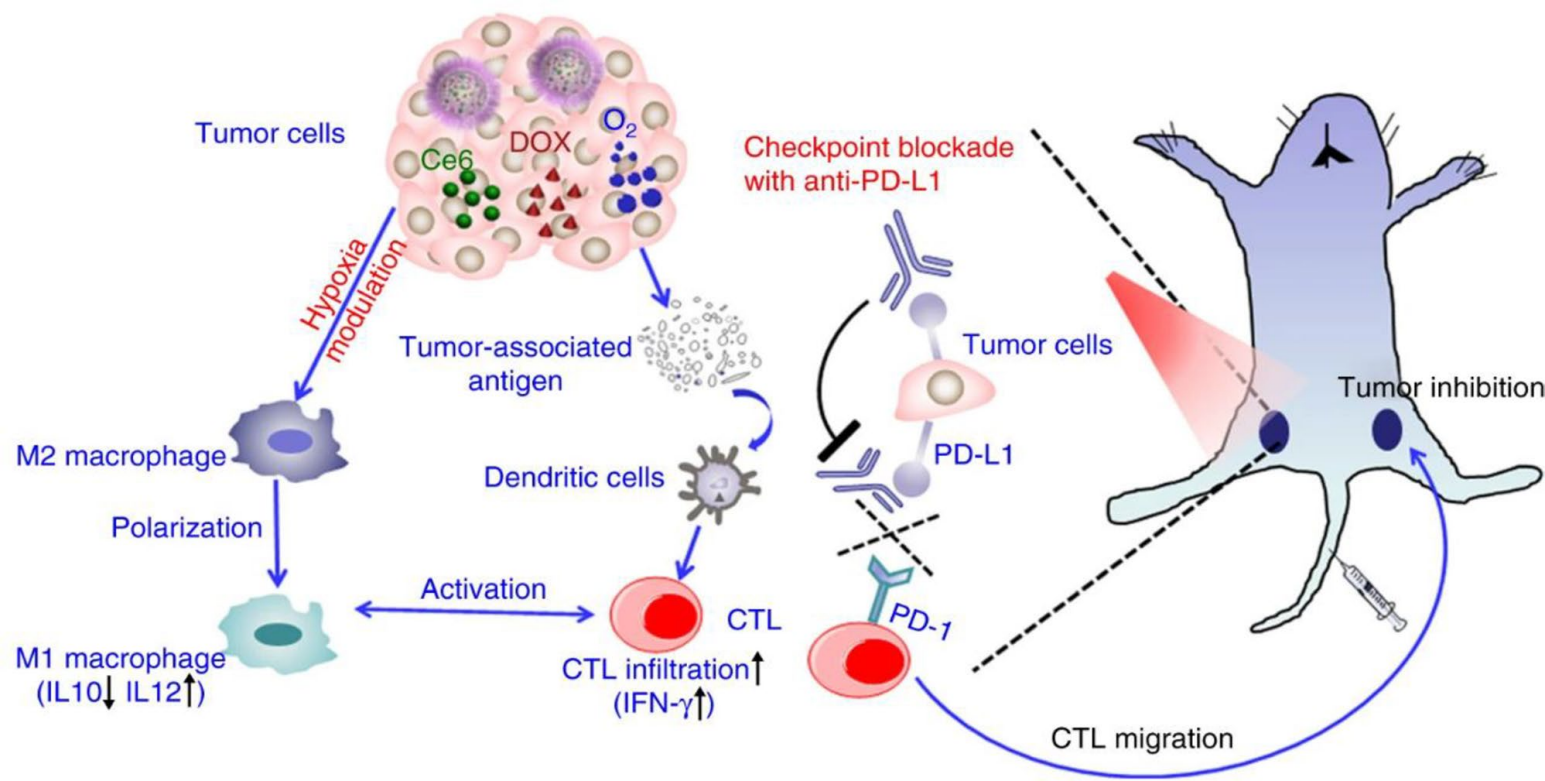
Schematic diagrams of the mechanism of anti-tumor immune responses induced by the combination of H-MnO₂-PEG/C&D and anti-PD-L1 Ab therapy. Reproduced with permission [46]

In addition to the role of MnO₂ NPs in triggering anti-tumor immune responses, chemotherapy and immunotherapy could be administered to achieve a synergistic therapeutic effect. Liu et al. designed H-MnO₂ nanoshells co-loaded with the photosensitizer Ce6 and the anti-tumor drug DOX. After injection into tumor-bearing mice, H-MnO₂ NPs accumulated in the tumors and degraded under acidic pH, leading to the generation of oxygen and the release of drugs. Subsequently, the 660-nm laser was irradiated with a tumor site to activate the Ce6 effects. Notably, there was considerable enhancement in macrophage infiltration within the tumors, accompanied by a reduced population of M2 TAMs. Additionally, there was a reduction in the population of other immunosuppressive immune cells and regulatory T cells, whereas increased infiltration of cytotoxic T lymphocytes (CTLs) was observed within the tumors. This outcome results from the TME modulation effect of H-MnO₂ NPs, which induces hypoxia attenuation, as well as from the recruitment and activation of anti-tumor immune cells through the cancer cell-killing effects of chemo-PDT therapy. Moreover, when PD-L1 checkpoint blockade, promising cancer immunotherapy, was simultaneously administered with H-MnO₂-PEG/C&D and laser treatment in mice bearing tumors on both flanks, a more pronounced delay in tumor growth was observed. Furthermore, CTL infiltration increased in the contralateral tumor that was not irradiated by the laser, indicating that combination therapy-induced cancer cell death leads to the release of tumor-associated antigens, which activate anti-tumor immune cells, such as macrophages, dendritic cells, and T cells. Adaptive immune responses were further improved in sensitive immunogenic tumors with mild hypoxia (Fig. 12) [46].

In cancer immunotherapy, MnO₂ NPs play multiple roles, including tumor-targeted drug delivery, alleviating hypoxia, creating an immunofavorable microenvironment, and inducing inflammatory responses through the activation of immune cells. Mn²⁺ is pivotal in immune function, and acts as a cofactor for key metalloenzymes such as manganese superoxide dismutase (MnSOD), glutamine synthetase, pyruvate carboxylase, and arginase [93, 94]. These enzymes are essential for immune cell activation of T-cells and macrophages, which are critical to immune responses. Mn²⁺ enhances both innate and adaptive immunity by promoting dendritic cell (DC) and macrophage maturation, improving antigen presentation, and augmenting the activation and differentiation of CD8⁺ T cells and natural killer (NK) cells. Additionally, Mn²⁺ increases the population of memory CD8⁺ T cells. When Mn²⁺ is combined with immune checkpoint inhibitors, it synergistically boosts antitumor immunity, and reduces the required dose of anti-PD-1 antibodies in mouse models. Mn²⁺ is also essential for effective tumor immune surveillance as Mn-deficient mice exhibit increased tumor growth, metastasis, and reduced tumor-infiltrating CD8⁺ T cells [95]. These immune-modulatory effects mediated through the cGAS-STING pathway underscore the critical role of Mn²⁺ in initiating and amplifying antitumor immunity [96]. Further studies are ongoing to combine MnO₂ NPs with other immunotherapies, such as co-loading anti-cancer antibodies, immune checkpoint blockade, adoptive cell transfer, and CAR cells to boost their anti-tumoral immune responses.

### H-MnO_2_ mediated nanovaccines

**Fig. 13:**
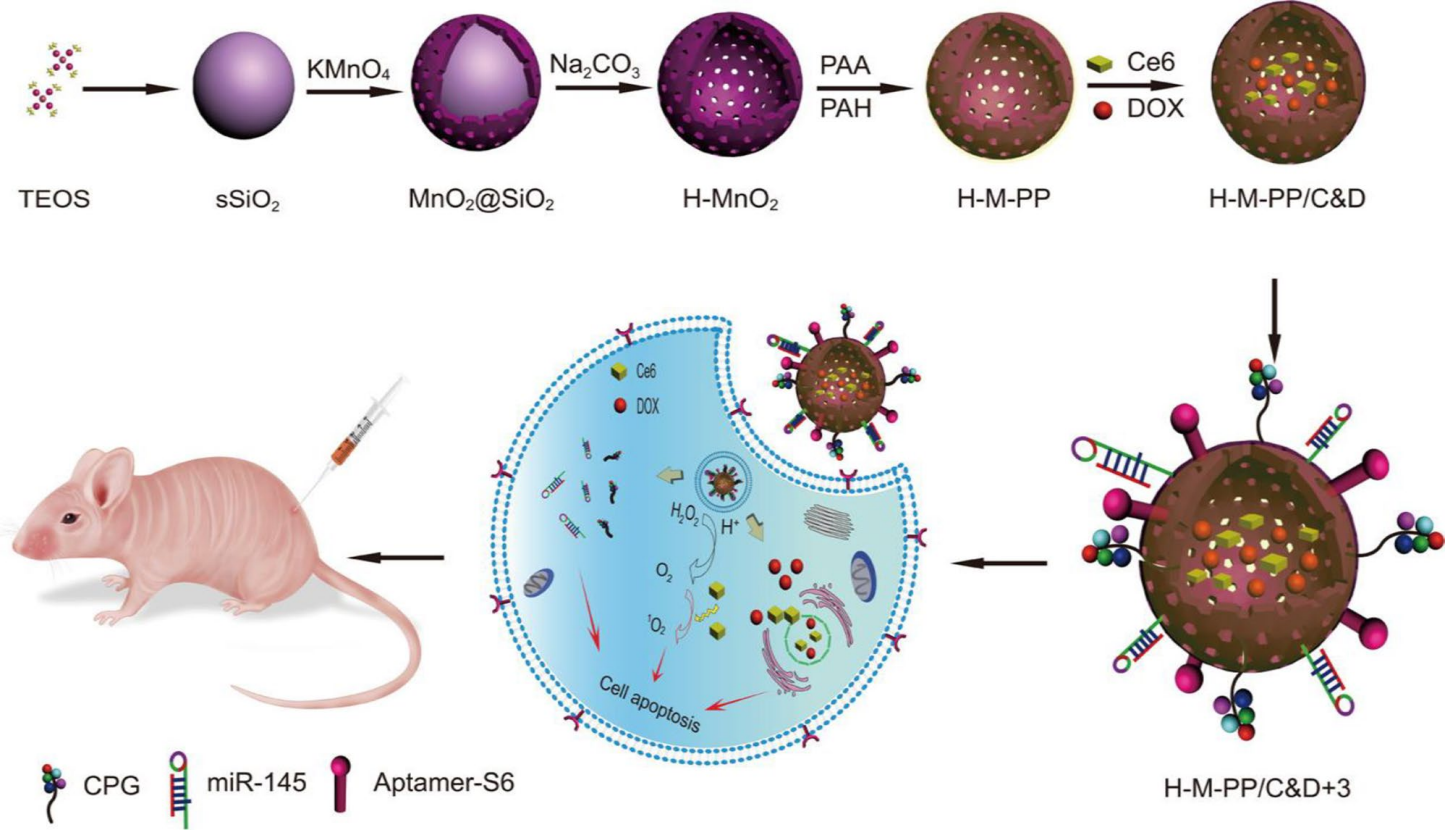
Schematic representation of the synthesis process and various functions of H-M-pp/C&D + 3 NPs loaded with functional genes, serving as synergistic nanovaccines for cancer immunotherapy. Reproduced with permission [12]

**Fig. 14:**
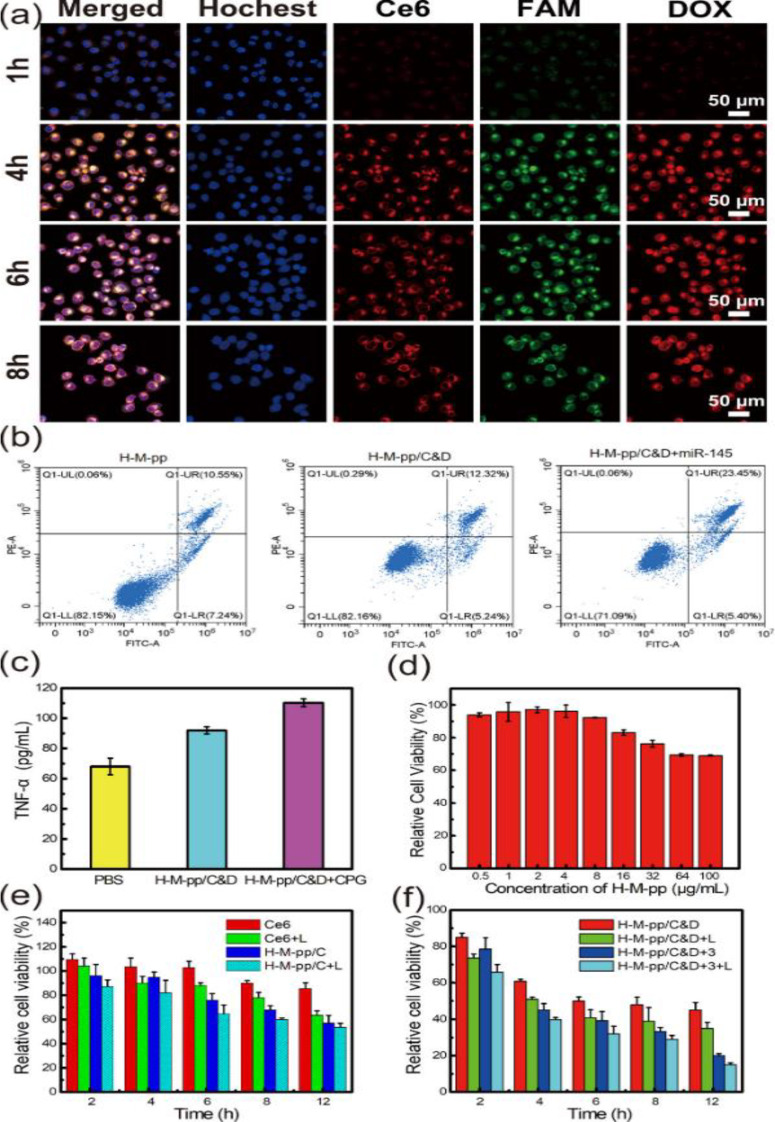
(**a**) CLSM images of A549 cells after treatment with H-M-pp/C&D + 3 nanoparticles. (**b)** The cell toxicities of H-M-pp, H-M-pp/C&D, and H-M-pp/C&D + miR-145 by flow cytotometry analysis. (**c)** TNF-α induced by DC2.4 cells incubated with PBS, H-M, and H-M-pp/C&D + CPG was determined by ELISA. **(d**) Cell viability at different concentrations of H-M-pp, (**e**) the effect of PDT on cell viability, and (**f**) cell viability under combined treatment with H-M-pp/C&D + 3 nanoparticles. Reproduced with permission [12]

Recently, Li et al. designed and synthesized novel intelligent H-MnO₂ NPs incorporating functional genes, aimed at developing them as nanovaccines [12]. These innovative nanovaccines offer significant potential in cancer immunotherapy by leveraging the unique properties of H-MnO₂ and their ability to deliver therapeutic genes effectively within the TME. The nanovaccines enabled the controlled release of targeted drugs in response to the TME, addressing tumor hypoxia while simultaneously enhancing the efficiency of PDT. This dual functionality improved tumor immune responses and facilitated a more effective combination therapy, resulting in significantly enhanced tumor treatment outcomes. To obtain the nanovaccines, H-MnO₂ was first coated with the anionic polymer PAA *via* the cationic polyelectrolyte PAH to form H-M-pp NPs. Subsequently, the photosensitizer Ce6 and anticancer drug DOX were encapsulated into the cavity of the H-M-pp NPs, producing H-M-pp/Ce6&DOX (H-M-pp/C&D). Finally, H-M-pp/C&D were conjugated with the amino-modified oligonucleotide CpG, S6-aptamers, and miR-145 through an amide coupling reaction to obtain H-M-pp/C&D + CpG/S6/miR-145 (H-M-pp/C&D + 3). The synthesis process and various functions of the H-M-pp/C&D + 3 NPs loaded with functional genes serving as synergistic nanovaccines for cancer immunotherapy are depicted in Fig. 13. This formulation exhibits enhanced water solubility and physiological stability, making it a promising candidate for improved therapeutic applications. Notably, the CpG oligonucleotide, an immunomodulatory adjuvant, is a toll-like receptor (TLR) agonist that triggers dendritic cells to enhance immunogenicity. The S6-aptamer in the H-M-pp/C&D + 3 nanovaccine confers exceptional specificity in targeting lung cancer cells. Reduced miR-145 expression in lung cancer cells promotes cell proliferation and metastasis and reduces apoptosis. Upon reaching the TME through the enhanced permeability and retention (EPR) effect and active targeting by the S6-aptamer, the H-MnO₂ nanoparticles react with H₂O₂ in the slightly acidic conditions of the TME, decomposing to produce Mn²⁺ and O₂. This decomposition enables the release of encapsulated drugs, while simultaneously improving the hypoxic environment of the tumor. The Mn²⁺ ions are absorbed by the body, mitigating potential tissue damage, and the generated oxygen enhances PDT, thereby amplifying the immune response against the tumor. This immunotherapy-based combination therapy holds significant promise as an effective and targeted approach for treating primary tumors and preventing tumor progression. To confirm the successful conjugation of the H-M-pp/C&D nanovaccine with CpG, S6-aptamer, and miR-145, three experiments were conducted (Fig. 14). First, cellular uptake of the S6-aptamer was assessed by labeling its 5′ end with FAM and incubating A549 cells with H-M-pp/C&D + S6. The cellular uptake was confirmed by confocal laser scanning microscopy (CLSM) images (Fig. 14a). To evaluate miR-145’s cytotoxic effects, A549 cells treated with H-M-pp, H-M-pp/C&D, or H-M-pp/C&D + miR-145 were analyzed by flow cytometry. The enhanced cytotoxicity was shown in A549 cells by the miR-145 upregulation (Fig. 14b). Immunostimulatory effects of H-M-pp/C&D + CpG were evaluated in DC2.4 cells. Significantly increased TNF-α secretion indicated a strong immune response (Fig. 14c). Cytotoxicity assays demonstrated that H-M-pp nanoparticles exhibited low toxicity at concentrations up to 100 µg/mL, and confirmed their biocompatibility (Fig. 14d). The PDT efficacy of H-M-pp nanoparticles loaded with Ce6 was also assessed. H-MnO₂-Ce6 showed higher cytotoxicity than free Ce6, both with and without laser exposure, due to better biocompatibility and increased Ce6 loading (Fig. 14e). Finally, the combination of PDT, chemotherapy, and immunotherapy with H-M-pp/C&D + 3 nanoparticles induced greater cytotoxicity over time, and highlighted the synergistic effects of multimodal treatment (Fig. 14f).

## Conclusions and future perspectives

In conclusion, this review highlights significant advancements in the synthesis and therapeutic applications of MONs. This review provides an overview of key synthetic methods, including thermal decomposition, potassium permanganate reduction, exfoliation, adsorption–oxidation, and hydro/solvothermal techniques. We delve into the preparation of MONs and H–MnO₂-based nanomaterials, discussing their chemical properties, surface modifications, and toxicity profiles. Furthermore, we highlight the notable applications of MONs in pH-responsive drug release, overcoming multidrug resistance (MDR), immunotherapy, and development of nanovaccines for synergistic cancer treatments. Despite these promising developments, several challenges remain for the clinical application of MONs. Although these materials offer significant advantages for cancer treatment, few clinical trials are currently underway, emphasizing the need for comprehensive safety evaluations. Although their synthetic routes have been extensively studied over the past decade, simpler and more efficient methods must be developed. The development of integrated nanotheranostic systems that seamlessly combine both diagnostic and therapeutic strategies remains a critical area of research. Moreover, establishing large datasets to assess the performance of MONs in treating different tumors is essential for guiding clinical practice.

Nevertheless, MONs exhibit significant potential in biomedicine, particularly in the targeted delivery of nanotherapeutics for treatment of cancer. We hope that this review provides a thorough understanding of MONs and their derivatives while offering key insights and guidance for the design of future MON-based nanoplatforms, ultimately facilitating their transition from laboratory research to clinical use.

## Data Availability

Data will be made available on request.
